# Cost-effectiveness of early empagliflozin initiation after acute myocardial infarction: a German statutory health insurance perspective

**DOI:** 10.1186/s12962-026-00830-0

**Published:** 2026-10-05

**Authors:** Priyanka Boettger, Eleni Nakou, Thomas Kinsley, Michael Buerke, Eva-Maria Wild, Samuel Sossalla

**Affiliations:** 1 Department of Internal Medicine I, Cardiology, Angiology and Intensive Care Medicine, Justus-Liebig-University, Giessen, Germany; 2 Department of Health Policy, London School of Economics and Political Science, London, UK; 3 Department of Cardiology, Galway University Hospital, Galway, Ireland; 4 Department of Internal Medicine II, Heart-Center-Südwestfalen, Cardiology, Angiology and Intensive Care Medicine, St. Marien-Krankenhaus, Siegen, Germany; 5 Faculty of Economics, Business Informatics and Business Law, University of Siegen, Siegen, North Rhine Westphalia Germany; 6 Cardiology Department, Royal Brompton and Harefield NHS Foundation Trust, London, UK; 7 Cardio-Pulmonary Institute (CPI), Justus Liebig University, Giessen, Germany

## Abstract

**Background and objective:**

Empagliflozin reduced heart failure (HF) hospitalizations after acute myocardial infarction (MI) in EMPACT-MI without a mortality benefit. We assessed whether HF morbidity prevention generates long-term economic value from the German statutory health insurance perspective.

**Methods:**

We developed a monthly cohort Markov model over a lifetime horizon comparing early empagliflozin plus standard care with standard care in post-MI patients at increased HF risk. The base case applied the EMPACT-MI hazard ratio of 0.77 for first HF hospitalization, assumed no direct mortality benefit, and modelled 24 months of treatment. Costs were in 2026 euros. The primary analysis covered 17.9 months; exploratory lifetime outcomes included quality-adjusted life-years (QALYs) and incremental cost-effectiveness ratios (ICERs).

**Results:**

Early empagliflozin gained 0.0315 QALYs at €1,418 incremental cost (ICER €45,100/QALY) and avoided 52.0 lifetime HF hospitalizations per 1000 patients. The probability of cost-effectiveness was 16.2%, 55.2%, and 79.7% at €30,000, €50,000, and €80,000/QALY, respectively. Price reductions of 25% and 50% lowered the ICER to €32,300/QALY and €19,500/QALY. The maximum annual price compatible with €30,000/QALY was €607. Results were most sensitive to the HF hospitalization effect, drug price, and baseline HF risk. A trial-anchored analysis using the EMPACT-MI placebo event and mortality rates yielded a similar ICER (€47,200/QALY).

**Conclusions:**

Early empagliflozin generated modest projected QALY gains, predominantly through modelled indirect survival gains, at higher lifetime costs.Economic value was conditional, depending on willingness-to-pay, net price, and absolute HF risk, with the most favorable profile at lower net prices and in HF-risk-enriched patients.

**Supplementary Information:**

The online version contains supplementary material available at https://doi.org/10.1186/s12962-026-00830-0.

## Key points for decision makers

• In a German statutory health insurance model without an assumed mortality benefit, early empagliflozin initiation after acute myocardial infarction generated modest quality-adjusted survival gains by reducing heart failure morbidity, with an ICER of approximately €45,100/QALY.

• Economic value was strongly dependent on drug price and baseline heart failure risk. Lower net prices and treatment of heart failure risk-enriched patients substantially improved cost-effectiveness.

• Because empagliflozin is not currently licensed specifically for heart failure prevention after acute myocardial infarction, these findings should be interpreted as an early modelling (early health technology assessment) exercise that quantifies potential long-term value rather than establishing cost-effectiveness.

**Supplementary Information:**

The online version contains supplementary material available at https://doi.org/10.1186/s12962-026-00830-0.

## Introduction

Acute myocardial infarction (MI) remains one of the most common and resource-intensive cardiovascular emergencies in Europe. Improved survival after MI has shifted attention to the subsequent development of heart failure (HF), a major driver of clinical, economic and humanistic burden [1, 2]. HF is among the most frequent reasons for hospitalization in Germany, accounting for an estimated €7.4 billion and 1.7% of disease-related expenditure, in 2020, mostly through inpatient care [3]. Because much of this burden arises from the transition from acute ischemic injury to chronic HF, preventing or delaying it matters not only for patient outcomes but for the value and sustainability of cardiovascular resource allocation.

Patients with left ventricular dysfunction, congestion during the index admission or other markers of high cardiovascular risk are particularly vulnerable to recurrent cardiovascular events and premature death [4, 5]. In these patients, post-MI HF is not a single acute complication but a chronic trajectory of recurrent admissions, long-term therapy and impaired quality of life. Because adverse left ventricular remodeling and neurohormonal activation begin early after infarction, there is a plausible therapeutic window for interventions that reduce congestion, attenuate remodeling or delay chronic HF development [6], and contemporary European guidelines emphasize early risk stratification and HF prevention after acute coronary syndromes [7, 8]. Pharmacological options have nonetheless remained limited: in PARADISE-MI, sacubitril/valsartan did not significantly reduce cardiovascular death or incident HF compared with ramipril after acute MI, underscoring the difficulty of modifying the early post-MI transition to HF [9].

Sodium–glucose cotransporter 2 (SGLT2) inhibitors have emerged as disease-modifying cardiovascular and renal therapies, reducing worsening HF events across reduced and preserved ejection fraction phenotypes and across high-risk cardiometabolic and renal populations [10–13]. This has raised interest in initiating SGLT2 inhibitors earlier in the cardiovascular disease trajectory, including after acute MI.

The clinical evidence for early SGLT2 inhibition after MI is still evolving [14, 15]. The EMMY trial showed favourable effects of empagliflozin on natriuretic peptides and echocardiographic remodeling markers after acute MI [16], and DAPA-MI showed cardiometabolic benefit in patients without prior diabetes or HF, although hard cardiovascular events were few [17]. EMPACT-MI evaluated empagliflozin in 6522 patients hospitalized for acute MI and at increased risk of HF: the primary composite of first HF hospitalization or all-cause death was not significantly reduced, but first and total HF hospitalizations were lower with empagliflozin, while all-cause mortality was similar between groups [18, 19]. This pattern of evidence creates a distinct health-economic question. A therapy without a statistically significant mortality benefit may still generate economic value if it prevents recurrent, resource-intensive morbidity; conversely, treating a large eligible post-MI population early would add substantial drug acquisition costs, and the absolute benefit per patient depends on baseline HF risk, treatment duration and persistence of effect. Prior cost-effectiveness findings for SGLT2 inhibitors in established heart failure will not reflect the early post-MI HF-prevention setting, because post-MI patients differ fundamentally in event rates, treatment timing and expected sources of benefit [20–22]. To our knowledge, no cost-effectiveness analysis of early empagliflozin, or of any SGLT2 inhibitor, initiated after acute MI has previously been published; the present study therefore provides the first economic evaluation of this strategy.

We conducted an early modelling (early health technology assessment) analysis to quantify the potential long-term value and price sensitivity of this strategy rather than to inform a specific reimbursement decision [23, 24]. The objective of this study was to evaluate the lifetime cost effectiveness of early empagliflozin initiation compared with standard care after acute MI in patients at increased risk of HF, from the perspective of the German statutory health insurance system. Using EMPACT-MI as the primary clinical evidence source and German cost inputs, we assessed whether a morbidity-driven treatment effect, reduced HF hospitalization without an assumed direct mortality benefit, can generate favourable long-term economic value, and identified the treatment-duration, drug-price and uncertainty assumptions under which this value changes.

## Methods

### Study design and reporting

We conducted a model-based cost–utility analysis comparing early empagliflozin initiation with standard care alone in patients hospitalized for acute MI and at increased risk of HF, from the perspective of the German statutory health insurance (SHI) system and including direct medical costs only. The study was designed and reported in accordance with the Consolidated Health Economic Evaluation Reporting Standards 2022 (CHEERS 2022) [23] (Supplemental Table 21), a full overview of the model and base-case assumptions is provided in Table 1. Because empagliflozin is authorized in the European Union for type 2 diabetes mellitus (T2DM), symptomatic chronic HF, and chronic kidney disease (CKD), but not specifically for HF prevention after acute MI, the analysis was framed as an early modelling (early health technology assessment) exercise assuming approval for early post-MI use.

**Table 1:** Model overview and base-case assumptions

Component	Base-case specification
Evaluation type	Model-based cost–utility analysis using a cohort state-transition Markov model
Reporting framework	Conducted and reported according to CHEERS 2022
Decision problem	Early empagliflozin initiation after acute myocardial infarction versus standard post-myocardial infarction care
Perspective	German statutory health insurance perspective
Cost categories	Direct medical costs only, including drug acquisition, cardiovascular hospitalizations, chronic disease management, and post-event care
Target population	Adults hospitalized for acute myocardial infarction and at increased risk of heart failure, reflecting EMPACT-MI eligibility criteria
Key eligibility features	Acute myocardial infarction within 14 days, newly developed left ventricular ejection fraction < 45%, signs or symptoms of congestion requiring treatment, or both, plus at least one additional enrichment factor
Main exclusion criteria reflected in model	Previous diagnosis of heart failure or planned sodium–glucose cotransporter 2 inhibitor use at baseline
Intervention	Empagliflozin 10 mg once daily initiated early after acute myocardial infarction, in addition to contemporary standard care
Comparator	Contemporary standard post-myocardial infarction care without systematic early sodium–glucose cotransporter 2 inhibitor initiation
Standard care	Guideline-based post-myocardial infarction care, including revascularization when indicated, antiplatelet therapy, lipid-lowering therapy, renin–angiotensin system inhibition, beta-blockers, mineralocorticoid receptor antagonists where indicated, cardiac rehabilitation, and risk-factor management
Delayed sodium–glucose cotransporter 2 inhibitor use after heart failure onset	Allowed in both arms where clinically appropriate, to reflect contemporary chronic heart failure care
Treatment duration	24 months in the base case
Treatment duration scenarios	18 months (reflecting EMPACT-MI follow-up), 5 years, and lifetime treatment
Cycle length	1 month
Time horizon	Lifetime, until death or age 100 years
Half-cycle correction	Applied to costs and health outcomes
Health states	Post-myocardial infarction without heart failure; post-myocardial infarction with heart failure; post-stroke without heart failure; post-stroke with heart failure; death
Acute events	Heart failure hospitalization, recurrent myocardial infarction, and stroke
Primary treatment-responsive event	First heart failure hospitalization
Main clinical effect	Reduction in first heart failure hospitalization while on treatment; hazard ratio and source reported in Table 2
Recurrent heart failure hospitalization effect	Not applied in the base case; total heart failure hospitalization rate ratio explored in scenario analysis (value and source in Table 2)
Mortality effect	No direct empagliflozin mortality benefit applied in the base case
Mortality scenario	Observed EMPACT-MI all-cause mortality hazard ratio explored in scenario analysis
Treatment-effect persistence	Treatment effect applied only while patients receive empagliflozin in the base case
Treatment-effect waning	Explored in structural scenario analyses
Health outcome measure	Quality-adjusted life-years and life-years
Utility approach	Health-state utilities assigned to post-myocardial infarction, chronic heart failure, post-stroke, and combined post-stroke with heart failure states
Direct empagliflozin utility benefit	Not applied in the base case
Acute disutilities	Applied during cycles with heart failure hospitalization, recurrent myocardial infarction, or stroke
Cost year	2026 euros
Drug cost	Public German pharmacy selling price used in the base case; confidential rebates explored through price-discount scenarios
Hospital event costs	Estimated using the German aG-DRG system and the 2026 federal base rate
Chronic heart failure costs	Annual maintenance costs applied separately from heart failure hospitalization costs to avoid double counting
Discount rate	3% annually for costs and health outcomes
Discounting scenarios	0%, 5%, and differential discounting explored in sensitivity analyses
Primary outcome	Incremental cost per quality-adjusted life-year gained
Secondary outcomes	Total costs, life-years, quality-adjusted life-years, heart failure hospitalizations avoided, recurrent myocardial infarctions, strokes, deaths, and incremental net monetary benefit
Willingness-to-pay interpretation	No official German QALY threshold assumed; results presented across willingness-to-pay values from €0 to €150,000 per QALY
Deterministic sensitivity analysis	One-way sensitivity analyses for key clinical, cost, utility, and treatment-duration parameters
Probabilistic sensitivity analysis	10,000 Monte Carlo simulations
Scenario analyses	Treatment duration, treatment-effect waning, recurrent heart failure hospitalization effect, delayed SGLT2 inhibitor use after heart failure onset, drug price discounts, alternative utility values, chronic heart failure cost assumptions, adverse events, adherence, and discounting
Threshold analyses	Annual empagliflozin net price, required price discount, heart failure hospitalization treatment effect, heart failure hospitalization cost, chronic heart failure cost, utility decrement, and treatment-effect duration
Value-of-information analysis	Expected value of perfect information estimated for the overall decision; population-level EVPI estimated over a 5-year German SHI decision horizon
Subgroup analyses	Age ≥ 65 years, type 2 diabetes or chronic kidney disease, no type 2 diabetes or chronic kidney disease, left ventricular ejection fraction < 35%, and congestion during index hospitalization
Budget impact analysis	Supplementary 5-year German statutory health insurance budget impact analysis with alternative uptake scenarios
Model validation	Face validity, internal validity, reproduction of EMPACT-MI placebo-arm outcomes, external validation against published post-myocardial infarction and heart failure literature, and technical code verification
Software	Model implemented in R

MI, myocardial infarction; HF, heart failure; SGLT2, sodium–glucose cotransporter 2; LV, left ventricular; QALY, quality-adjusted life-year; ICER, incremental cost-effectiveness ratio; SHI, statutory health insurance; HR, hazard ratio; DRG, diagnosis-related group; PSA, probabilistic sensitivity analysis; DSA, deterministic sensitivity analysis; EVPI, expected value of perfect information; INMB, incremental net monetary benefit; TECH-VER, technical verification checklist; EMPACT-MI and DAPA-MI, clinical trials; R, R statistical software

Abbreviations: aG-DRG, German diagnosis-related group; AMI, acute myocardial infarction; CHEERS, Consolidated Health Economic Evaluation Reporting Standards; CV, cardiovascular; DRG, diagnosis-related group; EVPI, expected value of perfect information; EVPPI, expected value of partial perfect information; HF, heart failure; ICER, incremental cost-effectiveness ratio; INMB, incremental net monetary benefit; LVEF, left ventricular ejection fraction; MI, myocardial infarction; QALY, quality-adjusted life-year; SGLT2, sodium-glucose cotransporter 2; SHI, statutory health insurance; T2DM, type 2 diabetes mellitus; WTP, willingness-to-pay

### Population, intervention, and comparator

The modelled population, intervention, comparator and standard-care regimen reflected EMPACT-MI and are specified in full in Table 1. Briefly, the population comprised adults hospitalized for acute MI and at increased risk of HF; the intervention was empagliflozin 10 mg once daily initiated early after MI in addition to contemporary standard care, and the comparator was the same standard care without systematic early SGLT2 inhibitor initiation. In the base case, empagliflozin was continued for 24 months [19, 25], with 18-month (reflecting EMPACT-MI follow-up), 5-year and lifetime durations evaluated in scenario analyses.

To reflect contemporary heart failure management, patients who developed clinically manifest chronic HF were assumed to become eligible for guideline-directed HF therapy, including SGLT2 inhibitors where clinically indicated. In the base case, delayed SGLT2 inhibitor initiation was assumed to occur after transition to the chronic HF health state and was modelled as reducing differences in long-term treatment exposure between study arms. Drug acquisition costs associated with delayed initiation were applied equally across treatment strategies after HF onset. No additional treatment benefit beyond that already captured through the prevention of HF events was applied, thereby avoiding double counting of SGLT2 inhibitor effects. Alternative assumptions regarding delayed initiation were explored in scenario analyses.

### Model structure

**Fig. 1:**
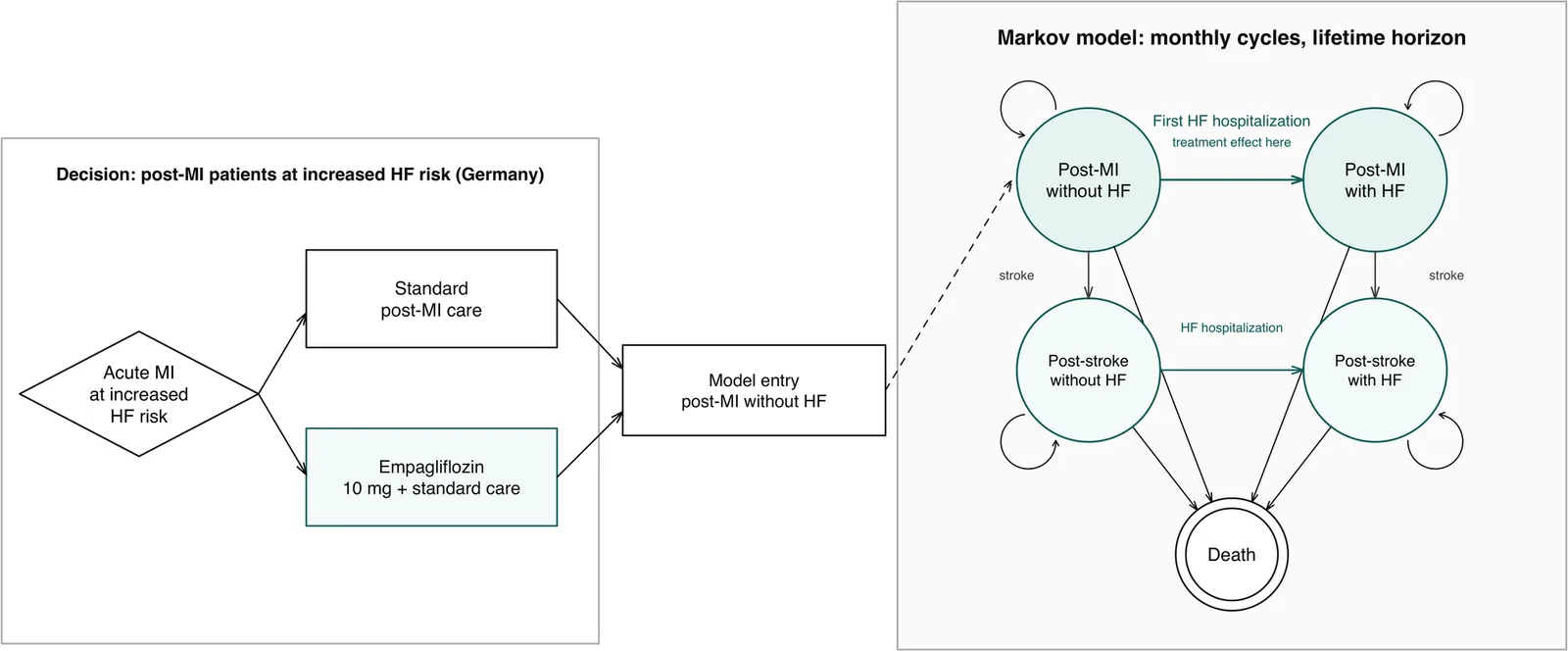
Model structure of the decision-analytic and state-transition model. Patients hospitalized with acute myocardial infarction and at increased risk of heart failure were allocated to early empagliflozin plus standard care or standard care alone. Following model entry, patients transitioned through monthly Markov cycles over a lifetime horizon. The treatment effect of empagliflozin was applied to first HF hospitalization, consistent with EMPACT-MI. Death was modelled as an absorbing state. Recurrent HF hospitalization, recurrent myocardial infarction, stroke, and their associated costs and utility decrements were incorporated as background events. HF, heart failure; MI, myocardial infarction

We developed a cohort state-transition Markov model with monthly cycles over a lifetime horizon (until death or age 100 years), with half-cycle correction. A Markov structure was chosen because the decision problem involved long-term extrapolation of the transition to chronic HF, recurrent cardiovascular events, and accumulation of costs and QALYs beyond trial follow-up. The model comprised five mutually exclusive health states, post-MI and post-stroke, each with and without HF, plus death, and three acute transition events: HF hospitalization (the primary treatment-responsive event), recurrent myocardial infarction, and stroke, each carrying event-specific costs, mortality, and utility decrements (Fig. 1). Stroke and recurrent myocardial infarction were included as background competing events because of their long-term impact on survival, costs, and quality of life. This deliberately parsimonious structure preserves transparency while capturing the clinically important transitions to chronic HF, recurrent events, and death. The model did not distinguish HF phenotypes, renal outcomes, incident diabetes, atrial fibrillation, or HF severity states as separate health states because EMPACT-MI did not provide sufficiently granular treatment-effect estimates for these pathways. Adding unsupported states would have increased structural uncertainty without improving evidentiary validity. Their potential influence was addressed through baseline-risk enrichment, utility and cost assumptions, subgroup analyses, and exploratory external-evidence scenarios. The full health-state and event implementation is shown in Fig. 1 and Supplemental Table 2.

### Clinical effectiveness and transition probabilities

Clinical efficacy inputs were derived from EMPACT-MI and its prespecified HF outcomes analysis [19, 25]. The hazard ratio for first HF hospitalization was applied as the main treatment effect (Table 2), while the total HF hospitalization rate ratio was explored in scenario analyses allowing an effect on recurrent HF hospitalizations. Although no direct treatment effect on recurrent heart failure hospitalizations was applied in the base case, the model nonetheless yields fewer recurrent hospitalizations, because reducing first hospitalizations leaves fewer patients in the chronic heart failure state and therefore at risk of subsequent events; this reduction is an indirect structural consequence rather than an independently modelled treatment effect. No direct mortality benefit was applied in the base case, consistent with EMPACT-MI; the observed all-cause mortality hazard ratio was explored in scenario analysis. Consistent with the neutral effect of SGLT2 inhibitors on stroke risk in cardiovascular outcome trials [26, 27], no treatment effect on stroke was applied, and recurrent myocardial infarction was likewise modelled without a direct treatment effect; both were therefore included as background competing events.

Baseline event probabilities were derived from placebo-arm EMPACT-MI event rates and published post-MI epidemiology, age- and sex-specific background mortality from German life tables, and model assumptions for excess mortality in chronic HF and stroke were informed by published cardiovascular literature. Full transition-probability derivations and treatment-effect implementation are provided in Supplemental Tables 2 and 3. Key clinical, cost, utility, and uncertainty parameters used in the model are summarized in Table 2.

Consistent with the German decision context, background mortality was modelled using age- and sex-specific mortality probabilities from the German period life table (Statistisches Bundesamt, Sterbetafel 2020/2022) [36], weighted to the sex distribution of the modelled cohort. Because patients after myocardial infarction experience mortality in excess of the general population, a constant post-MI excess-mortality hazard was added on top of the life-table background and calibrated so that modelled first-year all-cause mortality reproduced the approximately 3.7% observed in contemporary, guideline-treated German post-MI survivors in the OMEGA study [28]; excess mortality was thus layered on, rather than substituted for, the life-table background, avoiding double counting. This German-epidemiology configuration constitutes the base case. For the baseline first heart-failure-hospitalization risk we searched German statutory-claims and registry sources for a rate specific to a post-MI, reduced-ejection-fraction or congestion population comparable to EMPACT-MI eligibility; no sufficiently population- and endpoint-compatible German figure was identified, as available nationwide German cohorts are unselected and substantially older, with heart failure recorded at hospitalization far more frequently (left-ventricular heart failure in roughly 34–41% of infarctions) than the EMPACT-MI HF-hospitalization endpoint [29]. We therefore retained the randomized EMPACT-MI placebo first-HF-hospitalization rate for this parameter in the base case and used the broad German claims population only as a separate, explicitly labelled real-world/high-risk scenario. The relative treatment effect (first-HF-hospitalization hazard ratio 0.77) was taken unchanged from EMPACT-MI; German epidemiology informs the absolute baseline risks, not the randomized treatment effect. As a sensitivity and validation analysis we additionally report the fully trial-anchored configuration using the multinational EMPACT-MI placebo event and mortality rates (Table 5).

**Table 2:** Key model input parameters, uncertainty ranges, and sources

Parameter	Base-case value	Role in model	Sensitivity/scenario analysis	Source
**Treatment effects and structural assumptions**				
First HF hospitalization hazard ratio	0.77	Primary treatment effect on first HF hospitalization while receiving empagliflozin	Log-normal; 95% CI 0.60–0.98; threshold analysis	[18, 19]
Total HF hospitalization rate ratio	0.67	Scenario effect on recurrent HF hospitalizations	Scenario analysis; 95% CI 0.51–0.89	[19]
All-cause mortality hazard ratio	0.96	Not applied in base case; exploratory mortality scenario only	Scenario; 95% CI 0.78–1.19	[18]
Treatment duration	24 months	Base-case duration of empagliflozin treatment	18 months, 5 years, lifetime	[18]
Treatment-effect persistence	Effect restricted to treatment period	Base-case structural assumption	No residual effect, linear waning, persistent effect	Model assumption
**Costs (German SHI perspective)**				
Empagliflozin acquisition cost, €/year	860.75	Drug acquisition cost in intervention arm	25%, 50%, 75% price reductions; net price threshold	[30]
HF hospitalization cost, €/event	4,676	Acute cost of HF hospitalization	Low/high DRG scenarios; severe HF DRG scenario	[31]
Recurrent MI cost, €/event	8,000	Acute cost of recurrent MI	€4,700–€11,600; first-year post-MI cost scenario	[31, 32]
Acute stroke cost, €/event	7,572	Acute cost of stroke	€5,571–€12,001; first-year stroke cost scenario	[31, 32]
Chronic HF maintenance cost, €/year	1,157	Annual chronic HF cost excluding HF hospitalization	€700–€2,000; alternative total HF cost scenario	[32, 33]
Post-MI maintenance care, €/year	3,016	Annual cost in post-MI health states	€1,500–€4,500	[32]
Chronic post-stroke care cost, €/year	8,424	Annual post-stroke care cost	± 30%	[32]
**Utilities and disutilities**				
Post-MI utility	0.85	Utility for post-MI without chronic HF or stroke	0.78–0.90	[34, 35]
Chronic HF utility	0.76	Utility for chronic HF after MI	0.70–0.82	[34, 36]
Post-stroke utility	0.64	Utility for post-stroke health states	± 20%	[35, 37]
Post-stroke with HF utility	0.57	Combined post-stroke and chronic HF state	Multiplicative vs. additive decrement scenario	Model assumption (multiplicative combination)
Acute HF hospitalization disutility	0.194 (one cycle)	One-cycle disutility during HF hospitalization	± 20%	[22, 36]
Acute recurrent MI disutility	0.130 (one cycle)	One-cycle disutility during recurrent MI	± 20%	[34, 35]
Acute stroke disutility	0.290 (one cycle)	One-cycle disutility during acute stroke	± 20%	[35, 37]
**Mortality**				
Background mortality	Age- and sex-specific German life-table mortality	Background mortality risk	Alternative life-table scenario where applicable	[38]
Excess mortality with chronic HF	0.037 per year (model assumption)	Increased mortality in chronic HF states	Reported survival CIs or ± 20%	[39, 40]
Excess mortality after stroke	0.090 per year (model assumption)	Increased mortality after nonfatal stroke	Reported survival CIs or ± 20%	[41]
**Discounting**				
Discount rate	3% annually	Discounting of costs and QALYs	0%, 5%, differential discounting	[42]
First HF hospitalization rate (placebo)	4.7% at 17.9 months	Baseline first HF hospitalization risk	Rate uncertainty	EMPACT-MI
All-cause mortality (placebo)	5.5% at 17.9 months	Baseline mortality (background from German life tables)	± 20%	EMPACT-MI; Destatis
Recurrent HF hospitalization rate	Applied in chronic HF states	Determines recurrent HF hospitalization burden	± 20%	EMPACT-MI HF analysis

### Health outcomes and utilities

Health outcomes were life-years and QALYs, the latter combining survival time with health-state utilities and acute disutilities applied during cycles with HF hospitalization, recurrent myocardial infarction, or stroke. Utility estimates were drawn from published EQ-5D-based studies in myocardial infarction, HF, and stroke populations; where German EQ-5D value-set estimates were unavailable, European estimates were used and varied in sensitivity analyses, and overlapping chronic health states were combined multiplicatively. No independent empagliflozin-related quality-of-life benefit was applied in the base case, as EMPACT-MI did not provide utility data suitable for this analysis. Utility values, acute disutilities, and their uncertainty ranges are reported in Table 2 and Supplemental Table 4.

### Costs

Direct medical costs comprised empagliflozin acquisition, routine post-MI and chronic HF care, acute events (HF hospitalization, recurrent myocardial infarction, stroke), chronic post-stroke care, adverse events, and end-of-life care (Table 2). Hospital event costs were estimated from the 2026 German diagnosis-related groups excluding nursing staff costs (aG-DRG) catalogue and federal base rate, and empagliflozin from the public German pharmacy selling price for Jardiance 10 mg. Because confidential rebates and negotiated net prices are not publicly available, scenario analyses applied 25%, 50%, and 75% reductions to the annual acquisition cost while holding all other inputs constant, interpreted as plausible net-price conditions rather than observed reimbursement prices. Chronic HF maintenance costs excluded HF hospitalization costs to avoid double counting, and earlier estimates were inflated to 2026 euros using the German consumer price index where required. Cost inputs and uncertainty ranges are reported in Table 2, with detailed DRG mapping and alternative cost scenarios in Supplemental Table 5.

### Discounting, outcomes, and cost-effectiveness analysis

Costs and health outcomes were discounted at 3% annually; no discounting, 5% annual discounting, and differential discounting (3% for costs, 1.5% for effects) were evaluated in sensitivity analyses. Primary outcomes were total and incremental discounted costs and QALYs and the incremental cost-effectiveness ratio (ICER); secondary outcomes included life-years, HF hospitalizations avoided, recurrent myocardial infarctions, strokes, cardiovascular and non-cardiovascular deaths, and incremental net monetary benefit (Table 1).

### Uncertainty, scenario, and threshold analyses

Parameter uncertainty was assessed by one-way deterministic sensitivity analyses (inputs varied over reported 95% confidence intervals, or ± 20% where unavailable; Supplemental Table 12) and probabilistic sensitivity analysis with 10,000 Monte Carlo simulations (parameter distributions in Supplemental Table 6), summarized on a cost-effectiveness plane and cost-effectiveness acceptability curves (Supplemental Tables 13–14). To characterize price-dependent decision uncertainty, acceptability curves were additionally stratified by empagliflozin net-price scenario (current price and 25%, 50%, and 75% reductions). Because Germany has no official QALY-based threshold, willingness-to-pay values (€0–€150,000 per QALY) were interpreted as illustrative analytical reference points rather than reimbursement thresholds. Scenario analyses addressed structural and clinical uncertainty, treatment duration and waning, recurrent HF hospitalization effects, delayed SGLT2 inhibitor initiation after incident HF, drug price, utilities, chronic HF costs, adverse events, adherence, and discounting (Supplemental Table 11, Supplemental Fig. 4). Threshold analyses identified the net price, required price discount, treatment effect, event costs, utility decrement, and benefit duration at which conclusions changed (Supplemental Table 20).

As the modelled treatment effect derives from a component of a neutral primary endpoint, we additionally present a primary analysis restricted to the EMPACT-MI follow-up horizon (17.9 months), which over the trial period constitutes a cost-consequence comparison, and present the lifetime cost-utility analysis as an exploratory extrapolation. To characterise the dependence of the lifetime result on the key structural assumption that preventing an early heart failure hospitalization prevents or delays entry into the chronic heart failure state, we varied the proportion of prevented first heart failure hospitalizations that avoids chronic heart failure entry (0%, 25%, 50%, 75% and 100%), and decomposed the incremental QALY gain into survival (life-year) and quality-of-life components.

Expected value of perfect information (EVPI) was estimated; EVPI represents the maximum amount a decision maker would be willing to pay to eliminate all parameter uncertainty and thereby avoid the expected cost of an incorrect reimbursement decision, providing an upper bound on the value of further research rather than a measure of uncertainty itself. At each illustrative willingness-to-pay value, per-patient EVPI was calculated as the difference between the expected net monetary benefit under perfect information (the mean across probabilistic iterations of the maximum net benefit across strategies) and the expected net monetary benefit under current information (the maximum across strategies of the mean net benefit). Population-level EVPI was then estimated by multiplying per-patient EVPI by the discounted number of eligible SHI patients over a 5-year decision horizon. The annual eligible population was derived from hospitalized AMI cases in Germany, adjusted for SHI coverage and the proportion expected to meet EMPACT-MI-like high-risk eligibility criteria; alternative eligibility proportions were examined in sensitivity analyses. Exploratory external-evidence scenarios informed by DAPA-MI [17, 43] were conducted separately from the EMPACT-MI-based base case to assess how alternative post-MI SGLT2 inhibitor evidence might influence economic value estimates. These analyses were not intended as direct empagliflozin–dapagliflozin comparisons. Scenarios included a hard-outcome-only scenario replacing the EMPACT-MI first HF hospitalization effect with the DAPA-MI adjudicated estimate, with no mortality or recurrent HF hospitalization benefit; a cardiometabolic-benefit scenario additionally incorporating reduced new-onset T2DM and a modest health-status benefit; and lower-risk scenarios anchoring baseline HF hospitalization risk to DAPA-MI event rates.

### Subgroup and heterogeneity analyses

Prespecified subgroup analyses were conducted for clinically relevant high-risk groups (Table 1), using subgroup-specific baseline risks where available and the overall treatment effect where no credible treatment-effect heterogeneity was demonstrated. Accordingly, subgroup analyses were interpreted as risk-based value analyses reflecting differences in absolute event risk rather than as evidence of subgroup-specific treatment efficacy.

### Budget impact analysis

A supplementary 5-year budget impact analysis estimated the eligible population from annual German AMI hospitalizations and the proportion meeting EMPACT-MI risk criteria, under conservative, moderate, high, and full adoption scenarios (Supplemental Table 7, Supplemental Fig. 6). It included drug acquisition, avoided HF hospitalization, monitoring, and downstream chronic HF, recurrent myocardial infarction, and stroke costs; QALYs were not included.

### Model validation and software

Model validation comprised face, internal, external, and technical verification, guided by the TECH-VER verification checklist including clinical review of structure and assumptions, internal consistency checks, confirmation that modelled event rates reproduced the EMPACT-MI placebo-arm rates, comparison of long-term outputs with published post-MI and HF literature, and independent review of the R code (Supplemental Tables 8 and 10). The model was implemented in R; the planned reproducibility structure is given in Supplemental Table 9.

### Data Availability

Model input parameters, assumptions, and scenario definitions are provided in the manuscript and supplementary material. The model code is available upon reasonable request.

## Results

### Base-case cost-effectiveness results

In the base-case analysis, early empagliflozin initiation increased discounted quality-adjusted survival and lifetime costs compared with standard care (Table 3). Standard care generated 10.193 discounted life-years and 8.321 discounted QALYs at €47,520 per patient, compared with 10.221 life-years and 8.352 QALYs at €48,939 per patient with early empagliflozin. The incremental gain was 0.028 life-years and 0.0315 QALYs at an incremental cost of €1,418, yielding an ICER of approximately €45,100/QALY. Incremental net monetary benefit was negative at €30,000/QALY but became positive at €50,000/QALY and above. Early empagliflozin reduced the modeled lifetime burden of HF hospitalization, avoiding 52.0 total HF hospitalizations per 1000 patients, including 9.5 first and 42.5 recurrent HF hospitalizations; the reduction in recurrent hospitalizations arose indirectly, through fewer patients entering the chronic heart failure state rather than a modelled effect on recurrent events. Because no direct mortality benefit was assumed, life-year gains were modest and arose indirectly through delayed progression to chronic HF and lower downstream event burden.

**Table 3:** Base-case cost-effectiveness results, clinical outcomes, and discounted lifetime cost components

Outcome	Standard care	Early empagliflozin	Increment
**Cost-effectiveness outcomes**			
Discounted costs, €	47,520	48,939	1,418
Discounted QALYs	8.321	8.352	0.0315
Discounted life-years	10.193	10.221	0.028
ICER, €/QALY gained	—	—	45,100
INMB at €30,000/QALY, €	—	—	−474
INMB at €50,000/QALY, €	—	—	155
INMB at €80,000/QALY, €	—	—	1,099
INMB at €100,000/QALY, €	—	—	1,728
**Clinical outcomes per 1000 patients**			
First HF hospitalizations	342.4	333.0	−9.5
Recurrent HF hospitalizations	1140.0	1097.5	−42.5
Total HF hospitalizations	1482.4	1430.5	−52.0
**Discounted lifetime cost components**,** € per patient**			
Empagliflozin acquisition	0	1,610	1,610
Routine post-MI care	30,741	30,827	86
Chronic HF maintenance	2,190	2,091	−98
HF hospitalizations	4,932	4,720	−211
Recurrent MI events	2,443	2,450	7
Stroke events	1,074	1,077	2
Chronic post-stroke care	6,140	6,164	24
Total costs	47,520	48,939	1,418

Values are discounted over the lifetime horizon from the German statutory health insurance perspective. Clinical outcomes are reported per 1000 patients. Negative increments indicate fewer events or lower costs with early empagliflozin. HF, heart failure; ICER, incremental cost-effectiveness ratio; INMB, incremental net monetary benefit; MI, myocardial infarction; QALY, quality-adjusted life-year

### Primary trial-horizon analysis, structural uncertainty, and QALY decomposition

In an analysis restricted to the EMPACT-MI follow-up horizon (17.9 months), the trial-anchored, primary analysis, early empagliflozin incurred an incremental cost of approximately €1,157 per patient for the observed reductions in heart failure hospitalization, with a negligible incremental QALY gain (0.001). Over the trial horizon the evaluation therefore constitutes a cost-consequence comparison rather than a cost-utility analysis; the lifetime results should be interpreted as an exploratory extrapolation.

Because the lifetime QALY gain depends on the structural assumption that preventing an early heart failure hospitalization prevents or delays entry into the chronic heart failure state, we varied the proportion of prevented first hospitalizations that avoids chronic heart failure entry (Table 4). Assuming this proportion was 0% preventing only the acute hospitalization, with no durable divergence in disease trajectory the incremental QALY gain was 0.0002 and the ICER exceeded €7.0 million per QALY. As the proportion rose to 25%, 50%, 75% and 100% (the base case), the ICER fell to €189,600, €93,900, €61,500 and €45,100 per QALY, respectively. The lifetime cost-effectiveness of early empagliflozin is therefore entirely contingent on this structural assumption, for which EMPACT-MI provides no direct evidence.

**Table 4:** Sensitivity of the lifetime cost-effectiveness result to the proportion of prevented first heart failure hospitalizations that avoids entry into the chronic heart failure state

Proportion of prevented first HF hospitalizations avoiding chronic HF entry	Incremental cost (€)	Incremental QALYs	ICER (€/QALY)
0% (acute event only)	1,546	0.0002	6,967,365
25%	1,514	0.0080	189,600
50%	1,482	0.0158	93,900
75%	1,450	0.0236	61,500
100% (base case)	1,418	0.0315	45,100

Decomposing the lifetime incremental QALY gain (0.032) showed that approximately 76% (0.024 QALYs) derived from the modelled gain in life-years and only approximately 24% (0.008 QALYs) from improved quality of life. The survival component is a model-derived, indirect consequence of delayed progression to the higher-mortality chronic heart failure state, not a mortality benefit observed in EMPACT-MI

### Clinical and economic drivers of the base-case result

The incremental cost of early empagliflozin was primarily driven by drug acquisition. Discounted lifetime empagliflozin acquisition costs were €1,610 per patient. These costs were partly offset by fewer heart failure hospitalizations and lower chronic heart failure maintenance costs. The discounted reduction in heart failure hospitalization costs was €211 per patient, while chronic heart failure maintenance costs were reduced by €98 per patient. Costs related to recurrent myocardial infarction and stroke were similar between strategies because the base-case model did not apply a direct treatment effect of empagliflozin on these endpoints. Chronic post-stroke care costs were slightly higher in the early empagliflozin strategy because small gains in survival increased time at risk for chronic post-stroke care.

### Deterministic and probabilistic sensitivity analyses

**Fig. 2:**
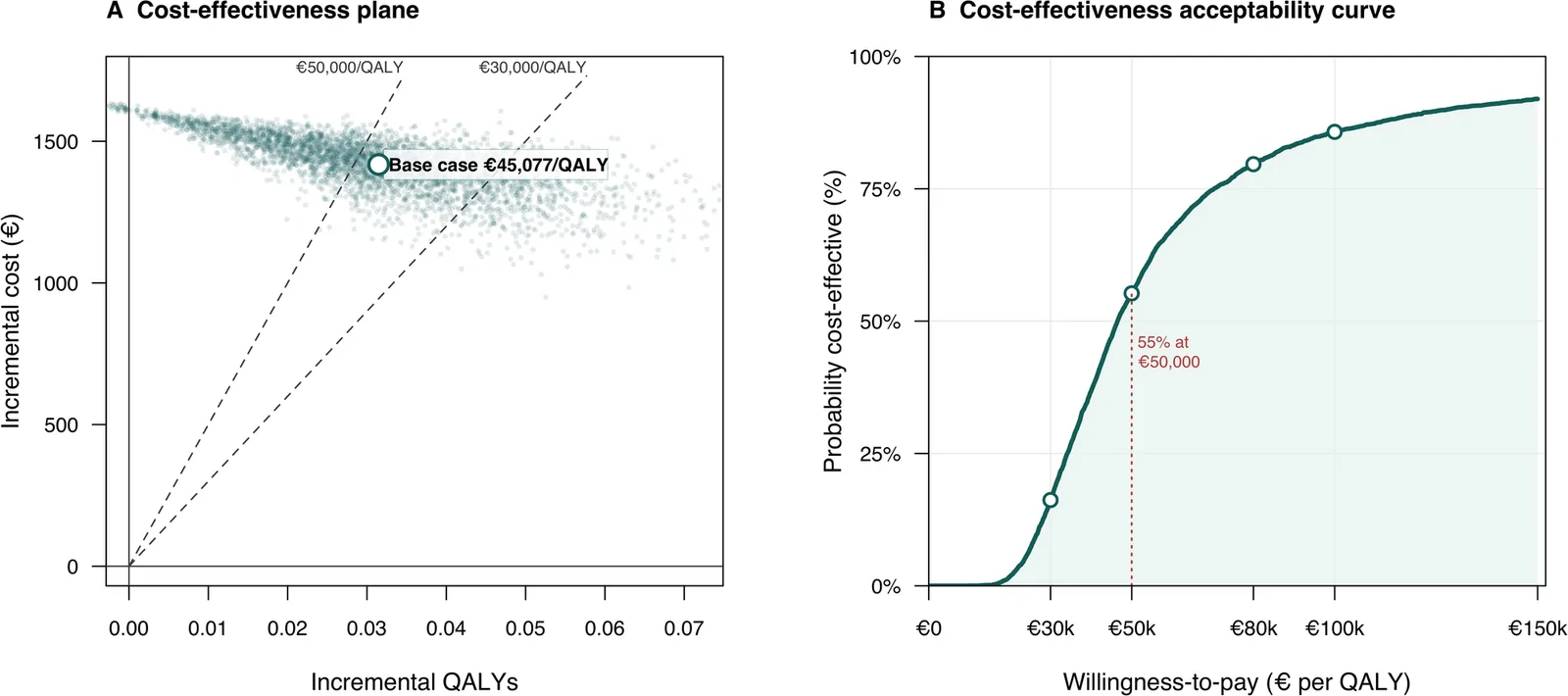
Probabilistic sensitivity analysis and cost-effectiveness acceptability curve. (**A**) Cost-effectiveness plane showing 10,000 probabilistic sensitivity analysis simulations for early empagliflozin versus standard care after acute myocardial infarction. Diagonal lines indicate illustrative willingness-to-pay values of €30,000, €50,000, and €100,000 per QALY; Germany has no formal QALY threshold. (**B**) Cost-effectiveness acceptability curve showing the probability that early empagliflozin is cost-effective across willingness-to-pay values. PSA, probabilistic sensitivity analysis; ICER, incremental cost-effectiveness ratio; QALY, quality-adjusted life-year

Probabilistic sensitivity analysis confirmed that early empagliflozin was associated with higher costs and modest gains in quality-adjusted survival, with substantial uncertainty around the magnitude of benefit. Early empagliflozin had a mean incremental cost of €1,425 and a mean incremental QALY gain of 0.0307, yielding a probabilistic mean ICER of approximately €46,500/QALY, consistent with the deterministic base-case estimate. The 95% simulation intervals were €1,197 to €1,596 for incremental costs and approximately 0.003 to 0.060 for incremental QALYs, reflecting uncertainty around the HF hospitalization treatment effect. The probability of cost-effectiveness was 16.2%, 55.2%, 79.7%, and 85.8% at illustrative willingness-to-pay values of €30,000, €50,000, €80,000, and €100,000/QALY, respectively. Per-patient EVPI was highest at €50,000/QALY (€269 per patient), indicating that decision uncertainty was greatest near this intermediate willingness-to-pay value. When scaled to the eligible German SHI population over a 5-year decision horizon, population-level EVPI was €52.9 million at €50,000/QALY, compared with €9.2 million at €30,000/QALY and €28.0 million at €80,000/QALY. In sensitivity analyses varying the proportion of AMI patients meeting EMPACT-MI-like high-risk eligibility criteria, population EVPI at €50,000/QALY ranged from €31.8 million to €74.1 million. The cost-effectiveness plane and cost-effectiveness acceptability curve are shown in Fig. 2.

**Fig. 3:**
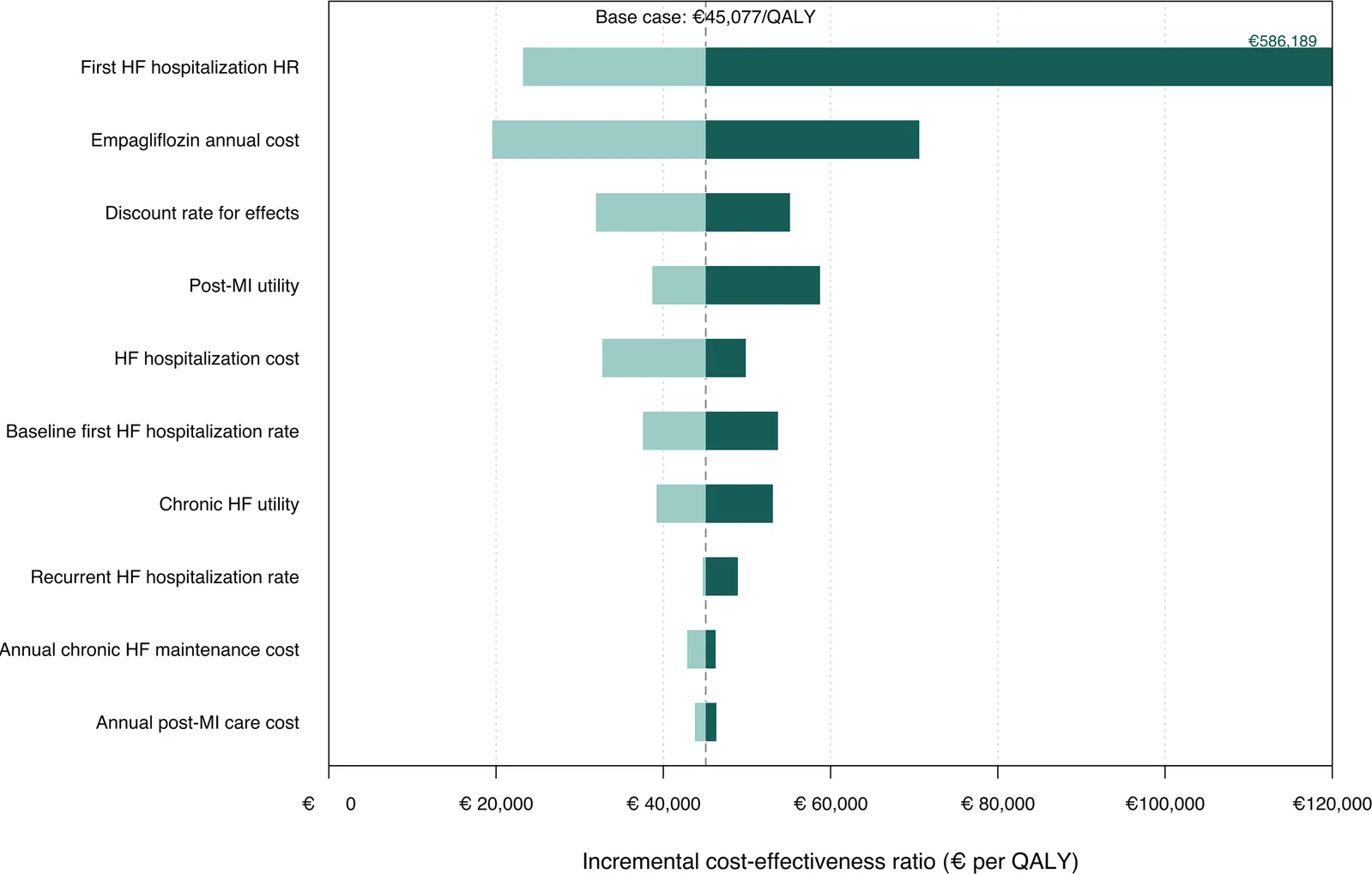
Deterministic sensitivity analysis of key model parameters. Tornado plot showing the effect of varying the ten most influential model parameters on the incremental cost-effectiveness ratio for early empagliflozin versus standard care after acute myocardial infarction. The dashed vertical line indicates the base-case ICER. Bars extending to the left and right show the ICER obtained under lower and upper parameter values, respectively. The treatment effect on first HF hospitalization was the dominant driver of uncertainty, followed by empagliflozin acquisition cost and health-state utility assumptions. HF, heart failure; HR, hazard ratio; ICER, incremental cost-effectiveness ratio; MI, myocardial infarction; QALY, quality-adjusted life-year

In one-way sensitivity analysis, the treatment effect on first HF hospitalization was the dominant driver of cost-effectiveness. Varying this hazard ratio across the EMPACT-MI 95% confidence interval shifted the ICER from approximately €23,200/QALY at the lower bound to €586,200/QALY at the upper bound. Other influential parameters were empagliflozin acquisition cost, post-MI utility, chronic HF utility, HF hospitalization cost, and the discount rate for health outcomes. Additional one-way analyses varying chronic HF and post-stroke excess-mortality hazards by ±20% yielded ICERs of €41,900–€49,300/QALY and €44,800–€45,300/QALY, respectively. The remaining deterministic sensitivity analysis is provided in the Supplement, and the ten most influential parameters are shown in Fig. 3.

### Scenario and price-threshold analyses

**Fig. 4:**
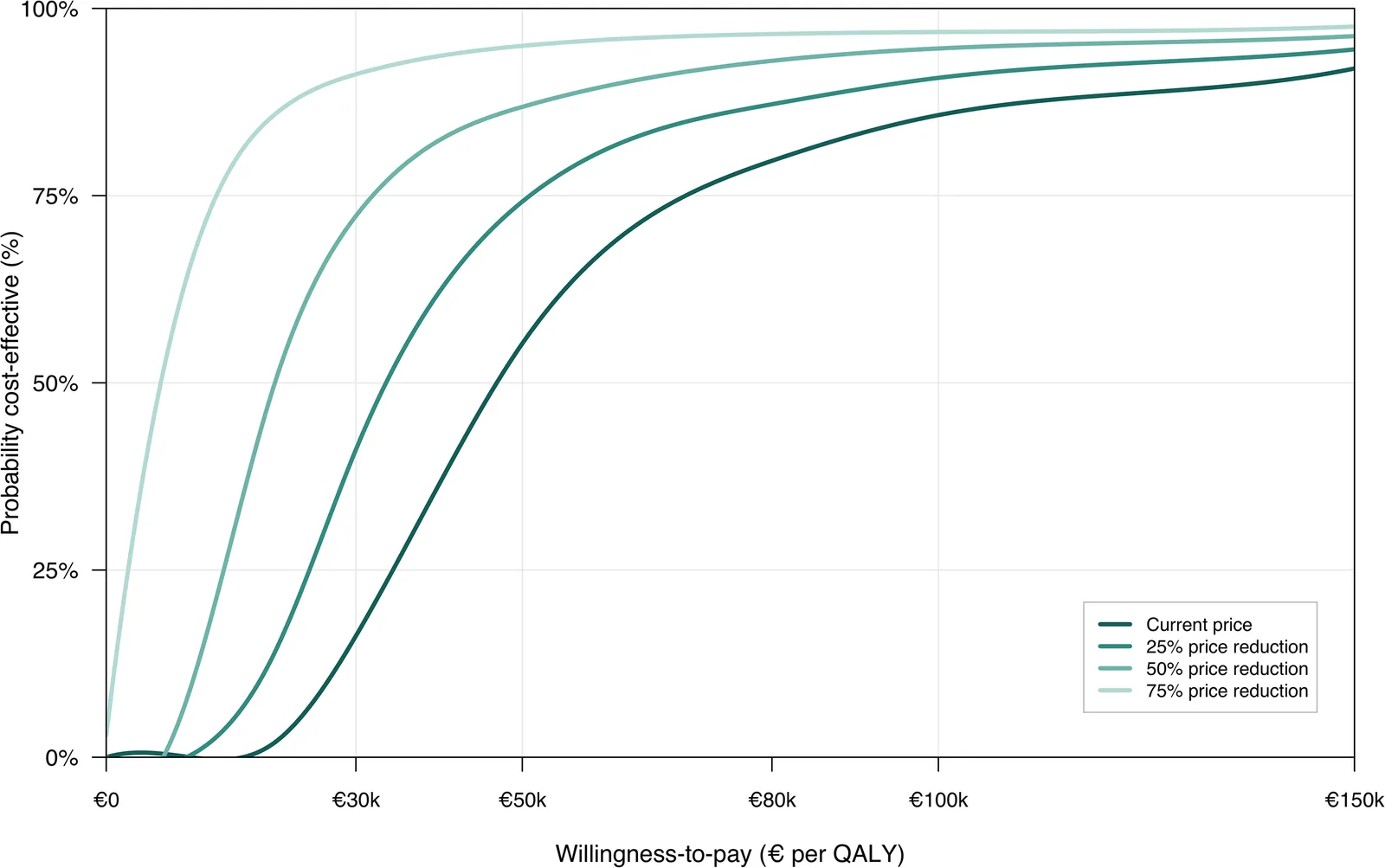
Impact of empagliflozin price reductions on the probability of cost-effectiveness. Cost-effectiveness acceptability curves (CEACs) derived from 10,000 probabilistic sensitivity analysis simulations showing the probability that early empagliflozin initiation after acute myocardial infarction is cost-effective across willingness-to-pay values from €0 to €150,000 per QALY. Curves are shown for the current public German pharmacy price and for hypothetical 25%, 50%, and 75% reductions in annual empagliflozin acquisition cost. Lower drug prices substantially increased the probability of cost-effectiveness across all willingness-to-pay values. Vertical dashed lines indicate illustrative reference values (€30,000, €50,000, and €100,000 per QALY). Germany has no formal cost-effectiveness threshold. CEAC, cost-effectiveness acceptability curve; QALY, quality-adjusted life-year

**Fig. 5:**
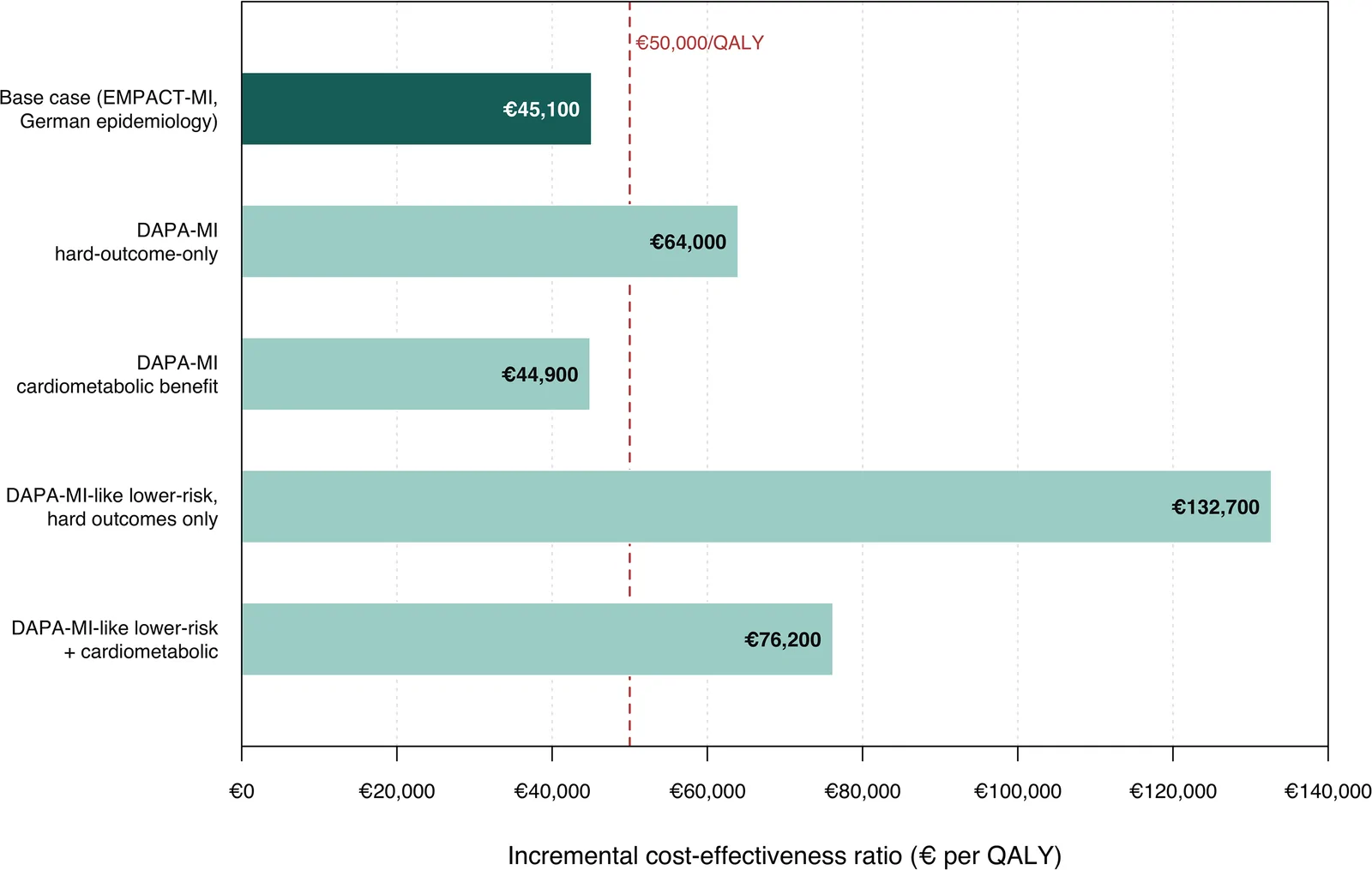
Cost-effectiveness of EMPACT-MI- and DAPA-MI-informed scenarios. Incremental cost-effectiveness ratios (ICERs) for the base-case EMPACT-MI scenario and exploratory scenarios informed by DAPA-MI. Scenarios included a hard-outcome-only approach based on adjudicated HF hospitalization, a cardiometabolic-benefit approach incorporating reduced new-onset type 2 diabetes and modest health-status improvement, and lower-risk post-MI population scenarios anchored to the lower event rates observed in DAPA-MI. The analysis illustrates the influence of baseline HF risk and broader cardiometabolic benefits on the estimated economic value of early SGLT2 inhibitor initiation after acute myocardial infarction. ICER, incremental cost-effectiveness ratio; HF, heart failure; MI, myocardial infarction; QALY, quality-adjusted life-year

Scenario, price-threshold, and selected decision-uncertainty results are summarized in Table 5. The ICER was most sensitive to treatment duration, drug acquisition cost, mortality assumptions, and the HF hospitalization treatment effect. A shorter treatment duration of 18 months yielded an ICER of €44,400/QALY, whereas 5-year and lifetime treatment increased the ICER to €49,100/QALY and €66,600/QALY, respectively. Applying the observed EMPACT-MI all-cause mortality hazard ratio improved the ICER to €28,600/QALY, although this scenario was exploratory because EMPACT-MI did not demonstrate a statistically significant mortality benefit. Drug price was a major determinant of value. Price reductions of 25%, 50%, and 75% lowered the ICER to €32,300/QALY, €19,500/QALY, and €6,700/QALY, respectively. At illustrative willingness-to-pay values of €30,000 and €50,000/QALY, the maximum compatible annual empagliflozin prices were €607 and €944 per patient-year. Price-dependent cost-effectiveness acceptability curves are shown in Fig. 4. In exploratory external-evidence scenarios informed by DAPA-MI, results varied according to whether only hard HF outcomes or broader cardiometabolic benefits were incorporated. These scenarios were not intended as direct empagliflozin–dapagliflozin comparisons. The hard-outcome-only scenario increased the ICER to €64,000/QALY, whereas adding reduced new-onset T2DM and a modest health-status benefit improved the ICER to €44,900/QALY. In DAPA-MI-like lower-risk populations, ICERs were higher, at €132,700/QALY with hard outcomes only and €76,200/QALY after incorporating cardiometabolic benefits (Fig. 5).

These findings indicate that economic value depends strongly on baseline HF risk, HF hospitalization reduction, and whether broader cardiometabolic benefits are valued. In exploratory risk-based subgroup analyses, the overall treatment effect was applied to subgroup-specific baseline HF risk derived from EMPACT-MI because no credible treatment-effect heterogeneity was demonstrated (P for interaction = 0.61). The placebo first-HF hospitalization incidence was 7.77 versus 4.05 per 100 patient-years in patients with versus without type 2 diabetes or chronic kidney disease [25]. This yielded a more favorable ICER of approximately €39,000/QALY (range €36,000–€42,000) in patients with type 2 diabetes or chronic kidney disease and a less favorable ICER of approximately €63,000/QALY (range €62,000–€65,000) in those without, reflecting greater absolute benefit at higher baseline risk rather than subgroup-specific treatment efficacy (Supplemental Table 15).

**Table 5:** Scenario, price-threshold, and decision-uncertainty analyses of early empagliflozin after acute myocardial infarction

Analysis	Incremental costs, €	Incremental QALYs	ICER or decision metric
Base case (German epidemiology)	1,418	0.0315	€45,100/QALY
**Scenario analyses**			
Treatment duration 18 months	1,081	0.0244	€44,400/QALY
Treatment duration 5 years	3,205	0.0653	€49,100/QALY
Lifetime treatment	7,937	0.1192	€66,600/QALY
Observed all-cause mortality HR 0.96 during treatment	1,561	0.0546	€28,600/QALY
EMPACT-MI trial-anchored (multinational placebo event and mortality rates)	1,417	0.0300	€47,200/QALY
Broad German claims, unselected higher-risk population	825	0.0359	€22,900/QALY
Low HF hospitalization cost	1,524	0.0315	€48,400/QALY
High HF hospitalization cost	1,313	0.0315	€41,700/QALY
No chronic HF maintenance cost increment	1,517	0.0315	€48,200/QALY
Larger HF utility decrement	1,418	0.0400	€35,500/QALY
**Exploratory external-evidence scenarios**			
DAPA-MI hard-outcome-only	1,478	0.0231	€64,000/QALY
DAPA-MI cardiometabolic-benefit	1,388	0.0309	€44,900/QALY
DAPA-MI-like lower-risk post-MI population, hard outcomes only	1,592	0.0120	€132,700/QALY
DAPA-MI-like lower-risk post-MI population plus cardiometabolic benefit	1,502	0.0197	€76,200/QALY
**Drug price analyses**			
25% drug price reduction	1,016	0.0315	€32,300/QALY
50% drug price reduction	613	0.0315	€19,500/QALY
75% drug price reduction	211	0.0315	€6,700/QALY
Maximum annual empagliflozin price at €30,000/QALY	—	—	€607/year
Maximum annual empagliflozin price at €50,000/QALY	—	—	€944/year
**Probabilistic and decision uncertainty analyses**			
Probability cost-effective at €30,000/QALY	—	—	16.2%
Probability cost-effective at €50,000/QALY	—	—	55.2%
Probability cost-effective at €80,000/QALY	—	—	79.7%
Probability cost-effective at €100,000/QALY	—	—	85.8%
EVPI at €50,000/QALY	—	—	€269 per patient
EVPI at €80,000/QALY	—	—	€142 per patient

Abbreviations: EVPI, expected value of perfect information; HF, heart failure; HR, hazard ratio; ICER, incremental cost-effectiveness ratio; MI, myocardial infarction; QALY, quality-adjusted life-year. Germany has no formal willingness-to-pay threshold for QALYs; threshold values are therefore used as illustrative decision benchmarks. The DAPA-MI hard-outcome-only scenario replaced the EMPACT-MI treatment effect on first HF hospitalization with the DAPA-MI estimate for adjudicated HF hospitalization and applied no direct mortality benefit or recurrent HF hospitalization effect. The DAPA-MI cardiometabolic-benefit scenario additionally incorporated reduced new-onset type 2 diabetes and a modest health-status benefit. The DAPA-MI-like lower-risk post-MI population scenarios additionally reduced baseline HF hospitalization risk to approximate the lower hard-event rates observed in DAPA-MI. These exploratory scenarios were not intended as direct comparisons between empagliflozin and dapagliflozin

### Model validation

The model closely reproduced the trial-period EMPACT-MI outcomes over the median follow-up of 17.9 months. The model predicted a first HF hospitalization risk of 4.6% in the standard-care arm and 3.5% in the early empagliflozin arm, compared with observed EMPACT-MI rates of 4.7% and 3.6%, respectively. Modelled all-cause mortality over the same period was approximately 5.6% in both arms, consistent with observed all-cause death rates of 5.5% in the placebo arm and 5.2% in the empagliflozin arm. Detailed validation results are provided in Supplemental Table 10.

## Discussion

### Principal findings

In this model-based economic evaluation from the perspective of the German statutory health insurance system, early empagliflozin initiation after acute MI in patients at increased risk of HF generated modest incremental health gains at higher lifetime costs. In the base case, early empagliflozin gained 0.0315 QALYs and 0.028 life-years at an incremental cost of €1,418 per patient, yielding an ICER of approximately €45,100/QALY. The absolute clinical benefit was modest, with 52.0 fewer lifetime HF hospitalizations per 1000 patients treated.

Economic value was driven primarily by reduced HF morbidity rather than by a direct mortality benefit. The base case deliberately assumed no direct mortality benefit, consistent with EMPACT-MI, in which empagliflozin did not significantly reduce the composite of first HF hospitalization or all-cause death, although first and total HF hospitalizations were reduced [18]. The model therefore estimated the value of preventing or delaying post-MI HF rather than that of a mortality-modifying post-MI therapy. At the German public pharmacy price, early empagliflozin was not cost saving, and cost-effectiveness was threshold-dependent and highly price-sensitive: the probability of cost-effectiveness was 16.2%, 55.2%, and 79.7% at €30,000, €50,000, and €80,000/QALY, respectively. Price reductions of 25% and 50% lowered the ICER to approximately €32,300/QALY and €19,500/QALY. These findings suggest that prevention of HF morbidity may confer economic value after MI, but that this value depends strongly on net price, baseline HF risk, and the decision-maker’s willingness to pay for incremental QALY gains.

### Clinical and economic interpretation

The clinical rationale for early post-MI HF prevention is strongest in patients with reduced LVEF, congestion, or other enrichment features, in whom registry and trial evidence indicate higher risks of HF events, recurrent hospitalization, and death [4, 5, 8, 18]. Adverse ventricular remodeling, neurohormonal activation, inflammation, and altered myocardial energetics begin early after infarction and may contribute to the transition from acute ischemic injury to chronic HF [6].

Pharmacological prevention of post-MI HF has nevertheless remained challenging. PARADISE-MI did not significantly reduce cardiovascular death or incident HF with sacubitril/valsartan compared with ramipril after acute MI in patients with reduced ejection fraction, pulmonary congestion, or both [9]. EMPACT-MI should be interpreted in this context: it did not establish empagliflozin as a mortality-reducing post-MI therapy, but it did provide evidence that early empagliflozin reduces HF events in a population enriched for post-MI HF risk [18, 19]. This distinction is central for economic evaluation. Mortality effects can generate large lifetime QALY gains, whereas prevention of nonfatal HF hospitalization produces smaller per-patient gains, particularly when absolute event rates are modest and treatment is applied broadly. In our model, avoided HF events generated measurable health gains, but the average QALY gain per treated patient remained modest. This pattern is consistent with economic principles in prevention: relative treatment effects translate into value through absolute event reduction, event disutility, downstream costs, and duration of benefit [23, 44].

### Relationship to the broader SGLT2 inhibitor evidence base and previous economic evaluations

These findings should be interpreted within the broader SGLT2 inhibitor evidence base. In established HFrEF, DAPA-HF and EMPEROR-Reduced showed that dapagliflozin and empagliflozin reduce worsening HF events and improve clinical outcomes [10, 12]. EMPEROR-Preserved and DELIVER extended this benefit to HF with mildly reduced or preserved ejection fraction [11, 13], and meta-analyses confirm reductions in HF hospitalization across HF phenotypes and high-risk cardiometabolic and renal populations [14, 45]. The post-MI setting is clinically and economically distinct. EMMY showed favorable effects of empagliflozin on NT-proBNP and remodeling markers after acute MI but was not powered for clinical outcomes [16]. DAPA-MI further illustrates how endpoint selection and baseline risk shape economic value after MI: dapagliflozin improved a hierarchical cardiometabolic endpoint in patients without prior diabetes or chronic HF, mainly through reduced new-onset T2DM, improved functional status, and weight reduction, but cardiovascular death and HF hospitalization rates were low and the composite of cardiovascular death or HF hospitalization was not reduced [17]. A later ejection-fraction analysis confirmed cardiometabolic benefit across LVEF strata without a clear effect on hard HF-related outcomes [46]. Our exploratory DAPA-MI-informed scenarios extended this evidence economically, showing less favorable value under hard-outcome-only assumptions and more favorable estimates when cardiometabolic benefits were included. Together, DAPA-MI and EMPACT-MI suggest that early post-MI SGLT2 inhibition may generate value through different pathways: HF-event prevention in higher-risk patients and broader cardiometabolic benefit in lower-risk populations. Our results are consistent with previous economic evaluations showing that SGLT2 inhibitor value depends on HF-event reduction, mortality assumptions, treatment price, and utility effects. Cost-effectiveness has generally been more favorable in HFrEF, where benefits include robust reductions in worsening HF and, in some models, survival gains or larger HF-event offsets [20, 21]. In HFpEF, value has been less favorable when no mortality benefit is assumed; Zheng et al. found empagliflozin to provide low economic value at US prices, driven by drug price, health-status benefit, and cardiovascular mortality assumptions [22]. The present analysis is closer to the HFpEF economic problem than to the HFrEF problem because EMPACT-MI demonstrated reduced HF events without an established mortality effect [18, 19]. However, post-MI HF prevention differs from established HF because treatment begins before HF is clinically manifest. Many treated patients will not develop HF, so even favorable relative effects may yield modest average benefit unless baseline HF risk is sufficiently high. This reinforces the importance of risk enrichment, consistent with prior economic work showing that baseline risk is a key determinant of cost-effectiveness for preventive cardiovascular therapies [47, 48].

### German reimbursement, pricing, and patient selection

The German reimbursement context requires careful interpretation. Germany does not use a formal QALY-based willingness-to-pay threshold within the AMNOG process; early benefit assessment focuses on added clinical benefit versus an appropriate comparator, followed by reimbursement-price negotiation with the National Association of Statutory Health Insurance Funds [24]. The present analysis should therefore not be interpreted as a direct German reimbursement recommendation, but as complementary evidence on long-term value, price sensitivity, affordability, and decision uncertainty under a hypothetical post-MI HF-prevention indication.

At the current public pharmacy price, early empagliflozin was unlikely to be economically compelling through cost offsets alone. Drug acquisition costs outweighed savings from avoided HF hospitalizations and reduced chronic HF costs, even though German cardiovascular event costs are substantial and concentrated in the acute hospitalization and early post-event period [32]. The price-threshold analysis is therefore central: at illustrative values of €30,000 and €50,000/QALY, the maximum compatible annual empagliflozin prices were approximately €607 and €944 per patient-year, respectively. Because confidential rebates and negotiated net prices are not publicly observable, public list-price analyses may not reflect true payer costs; nevertheless, threshold analyses identify the net-price range in which early post-MI use becomes economically more attractive.

The clinical and economic case is likely to be strongest in patients at higher absolute risk of post-MI HF. EMPACT-MI enriched for such risk by enrolling patients with newly reduced LVEF, congestion requiring treatment, or both, plus at least one additional enrichment factor [18]. The findings should therefore not be read as support for unselected post-MI use. A more plausible value-based approach would focus on patients with clear HF-risk markers, including reduced LVEF, congestion during the index hospitalization, diabetes, renal dysfunction, elevated natriuretic peptides, previous MI, atrial fibrillation, or incomplete revascularization [7, 8, 49].

The contemporary HF guideline context also affects the comparator. SGLT2 inhibitors are now recommended as foundational therapy across much of the HF spectrum [7, 47]. The relevant comparison is therefore not lifelong SGLT2 inhibitor use versus none, but early post-MI initiation versus delayed initiation after clinically manifest HF. In patients with T2DM, CKD, or established HF, SGLT2 inhibitors may already be indicated because of cardiometabolic, renal, and HF benefits [7, 47–50]. In patients without these indications, the incremental clinical and economic justification depends more specifically on the post-MI HF-prevention effect observed in EMPACT-MI and supported by the post hoc analyses of Fioretti et al. [18, 25].

### Uncertainty, strengths, and limitations

Decision uncertainty was substantial. At €50,000/QALY, the probability that early empagliflozin was cost-effective was approximately 55%, and per-patient EVPI was highest at €269, indicating that the intervention lies near an intermediate decision boundary rather than being clearly high or low value. When scaled to the eligible German SHI population, population-level EVPI was also greatest at this value, supporting the potential value of further evidence generation. The main drivers of uncertainty were the treatment effect on HF hospitalization, drug price, treatment duration, HF-related utility loss, and assumptions about long-term persistence of benefit. This pattern is expected because the base case did not include a direct mortality benefit or independent health-status effect and therefore relied primarily on the magnitude and durability of HF hospitalization reduction. Longer follow-up of EMPACT-MI, pooled analyses across post-MI SGLT2 inhibitor trials, individual patient data meta-analysis, and German claims-based studies would help clarify absolute event reduction, recurrent HF event rates, long-term resource use, treatment uptake after HF onset, and subgroup-specific value [17–19, 25].

This analysis has several strengths. To our knowledge, it is the first cost-effectiveness analysis to evaluate early empagliflozin initiation after acute MI and the first economic evaluation anchored directly to EMPACT-MI. The model was therefore closely aligned with the most relevant randomized evidence, with EMPACT-MI informing early empagliflozin use after acute MI and the prespecified HF outcomes analysis informing first and total HF hospitalization effects [18, 19]. The base case excluded any direct mortality benefit, thereby reducing the risk of overstating value. The analysis adopted a German statutory health insurance perspective, used German drug prices, DRG-based hospitalization costs, and national cardiovascular cost inputs where available [32], and explored key clinical, structural, and economic uncertainties through treatment-duration scenarios, delayed SGLT2 inhibitor initiation after HF diagnosis, drug-price thresholds, deterministic and probabilistic sensitivity analyses, and EVPI, consistent with good practice for model-based economic evaluation [23, 44]. The model also reproduced short-term EMPACT-MI event rates closely, supporting the internal consistency of the trial extrapolation.

Several limitations should be acknowledged. First, the model relied on aggregate published trial data rather than individual patient data, limiting the ability to model patient-level heterogeneity, competing risks, treatment discontinuation, recurrent event histories, and risk-based subgroup selection; individual patient data would allow more precise estimation of absolute benefit in high-risk subgroups [48]. Second, lifetime extrapolation beyond the median EMPACT-MI follow-up of 17.9 months was necessary to capture downstream HF and cardiovascular consequences, but introduced uncertainty regarding treatment-effect persistence, post-treatment waning, and long-term HF progression [23, 44]. Third, the exclusion of a direct mortality benefit is consistent with the neutral EMPACT-MI result, but may underestimate value if future evidence demonstrates cardiovascular or all-cause mortality benefit after MI; conversely, exploratory mortality scenarios should be interpreted cautiously because the observed mortality effect was not statistically significant [18]. Utility estimates were not derived directly from EMPACT-MI and may not fully reflect German post-MI preferences, which is relevant because incremental QALY gains were modest and utility assumptions materially influenced the ICER [11, 22]. Cost inputs were based on German public prices and tariff sources rather than patient-level claims data; public pharmacy prices may differ from net statutory health insurance costs after rebates, and DRG-based event costs may not capture outpatient care, rehabilitation, long-term care, productivity losses, or informal care [32]. Generalizability to other payer settings may therefore be limited. Finally, the simplified health-state structure did not distinguish HF phenotypes after MI or explicitly model renal outcomes, new-onset diabetes, atrial fibrillation, or cardiometabolic outcomes as separate states. This preserved transparency but may underestimate benefits suggested by DAPA-MI, including effects on new-onset diabetes, body weight, atrial fibrillation, and functional status [46]. Because empagliflozin is not currently licensed specifically for HF prevention after acute MI, the analysis should be interpreted as an early modelling (early health technology assessment) exercise.

An important limitation concerns the strength of the clinical evidence underpinning the modelled treatment effect. EMPACT-MI did not meet its primary composite endpoint of first heart failure hospitalization or all-cause death, and no mortality benefit was demonstrated; the reduction in first and total heart failure hospitalizations that anchors the model derives from a component of a neutral primary endpoint and should therefore be interpreted with caution [18, 19]. DAPA-MI likewise did not establish a benefit on major adjudicated cardiovascular outcomes [17]. Because the entire chain of downstream modelled benefit reduced progression to chronic heart failure, avoided hospitalizations, gains in life-years and QALYs, and ultimately the cost-effectiveness estimate, rests on this single, statistically fragile effect, the present findings should be regarded as exploratory and hypothesis-generating rather than as confirmation of established long-term cost-effectiveness. The analysis is best interpreted as a conditional economic assessment that quantifies the value early empagliflozin would generate if the observed reduction in heart failure hospitalization reflects a true and durable effect, and that identifies the price, baseline-risk and effect-duration conditions under which that value would or would not be realized.

A second limitation concerns the base-case treatment duration and its lifetime extrapolation. In the base case, empagliflozin was continued for 24 months and the treatment effect was applied only while on treatment, with no assumed post-treatment persistence. Because this base-case duration exceeds the 17.9-month median follow-up of EMPACT-MI [18], it reflects an assumption of continued real-world treatment beyond trial follow-up; we therefore also report an 18-month, trial-anchored scenario as a shorter, directly evidence-based alternative, which yielded a similar ICER (€44,400 per QALY). More fundamentally, because preventing an early heart failure hospitalization delays entry into the higher-cost, higher-mortality chronic heart failure state, the model generates lifetime differences in costs, hospitalizations and survival that exceed those observed within trial follow-up. We emphasize that these downstream gains, including the modest 0.028 incremental life-years, arise from the structural assumption that a time-limited reduction in incident heart failure produces durable divergence in disease trajectory, rather than from directly observed trial data; EMPACT-MI provides no evidence that an early reduction in heart failure hospitalization translates into permanent reductions in heart failure progression, mortality or quality-of-life loss. Decomposing the incremental QALY gain clarifies this distinction: approximately three-quarters of the 0.0315 QALY gain reflects a model-derived, indirect survival gain additional life-years accrued by delaying entry into the higher-mortality chronic heart failure state rather than a mortality benefit observed in EMPACT-MI, with the remaining quarter arising from avoided time in the lower-utility heart failure state.

A further consideration concerns the source of baseline event rates. Following reviewer guidance, background mortality in the primary analysis was anchored to German life tables and calibrated to German post-MI mortality, strengthening national relevance. For the baseline first-HF-hospitalization risk, however, no German claims or registry source sufficiently matched the EMPACT-MI-eligible population (post-MI with reduced ejection fraction or congestion) in both population and endpoint definition; available nationwide German cohorts are unselected and substantially older, with heart failure recorded at hospitalization far more frequently than the trial’s HF-hospitalization endpoint, so applying their raw event rates would misrepresent the trial-eligible population and overstate the absolute number of preventable hospitalizations [29]. We therefore retained the randomized EMPACT-MI placebo rate for this parameter and present the German claims population only as a real-world/high-risk scenario, in which reflecting the higher absolute event burden the ICER was more favourable (approximately €22,900/QALY). Conversely, the fully trial-anchored analysis using EMPACT-MI placebo mortality yielded a closely similar ICER (approximately €47,200/QALY), indicating that the German mortality recalibration did not materially change the base-case conclusion.

### Conclusions

Early empagliflozin initiation after acute MI in patients at increased risk of HF modestly improved quality-adjusted survival and reduced HF hospitalizations, but increased lifetime costs from the German statutory health insurance perspective. In a base case without an assumed direct mortality benefit, the ICER was approximately €45,100/QALY. Economic value was driven primarily by prevention of HF morbidity and was highly sensitive to drug price, baseline HF risk, and the treatment effect on HF hospitalization. These findings suggest that early post-MI empagliflozin may provide value under a potential HF-prevention indication, particularly at lower net prices or in HF-risk-enriched patients, while highlighting substantial decision uncertainty and the need for further evidence on long-term outcomes, patient selection, quality of life, and German real-world costs. Given the neutral primary endpoint of EMPACT-MI, these findings should be regarded as exploratory and hypothesis-generating; they identify the conditions, notably lower net price and higher baseline heart failure risk, under which early empagliflozin could offer economic value, rather than establishing cost-effectiveness.

## Supplementary Information

Below is the link to the electronic supplementary material.

Supplementary Material 1

## Data Availability

All data used in this study were derived from published aggregate clinical evidence and publicly available or licensed German cost sources. The model inputs, assumptions, parameter ranges, and scenario definitions are provided in the manuscript and supplementary materials. No individual patient-level data were used. Additional model outputs and the analytic code are available from the corresponding author upon reasonable request, subject to restrictions applicable to proprietary cost catalogues and licensed data sources.

## Code Availability

The analytic model code will be made available from the corresponding author upon reasonable request and may be deposited in an open repository if required by the journal or as a condition of publication, subject to restrictions related to proprietary cost catalogues and licensed cost inputs.

## Declarations

### Declaration of AI-assisted technologies

During manuscript preparation, Microsoft Copilot was used to assist with language polishing and grammatical refinement. No AI tool was used for data analysis, interpretation, or generation of scientific content. The authors critically reviewed and approved the final manuscript.
