# Supplementary material for: Cost-effectiveness of early empagliflozin initiation after acute myocardial infarction: a German statutory health insurance perspective
Source: Cost Eff Resour Alloc. 2026 Oct 5;24:123. doi: 10.1186/s12962-026-00830-0 (PMC13637204; doi:10.1186/s12962-026-00830-0)
Supplement: Supplementary file 1 [file 12962_2026_830_MOESM1_ESM.docx]

**Supplemental Appendix**

**Cost-Effectiveness of Early Empagliflozin Initiation After Acute Myocardial Infarction: A German Statutory Health Insurance Perspective**

This supplemental appendix provides technical model details, input tables, additional sensitivity analyses, validation outputs, and supplemental figures. To minimize overlap with the main manuscript, base-case results and primary interpretation are not repeated except where needed to contextualize supplemental analyses.

# Supplemental Methods

## Model overview and core assumptions

The analysis used a cohort state-transition model with monthly cycles. Patients entered the model in the post-myocardial infarction state without clinically manifest heart failure. During each cycle, patients could remain in their current state, develop chronic heart failure, experience stroke, experience acute transition events, or die. Heart failure hospitalization and recurrent myocardial infarction were implemented as transition events with acute costs and utility decrements; stroke was modelled as both an acute event and a chronic post-stroke health state.

**Supplemental Table 1.** Model overview and core assumptions.

| **Component** | **Specification** |
| --- | --- |
| Decision problem | Early empagliflozin initiation after acute myocardial infarction versus contemporary standard post-myocardial infarction care |
| Perspective | German statutory health insurance (SHI); direct medical costs only |
| Population | Adults hospitalized for acute myocardial infarction and at increased risk of heart failure, aligned with EMPACT-MI |
| Intervention | Empagliflozin 10 mg once daily plus standard care |
| Comparator | Standard post-myocardial infarction care without systematic early SGLT2 inhibitor initiation |
| Cycle length | 1 month |
| Time horizon | Lifetime, until death or age 100 years |
| Discounting | 3% annually for costs and health outcomes in the base case |
| Treatment duration | 18 months in the current base-case model output; alternative durations examined in scenario analyses |
| Direct mortality benefit | Not applied in the base case |
| Primary treatment-responsive event | First heart failure hospitalization/incident clinically manifest heart failure |
| Recurrent HF hospitalization effect | Examined in scenario analysis using total HF hospitalization rate ratio |
| Direct empagliflozin utility benefit | Not applied in the base case |

AMI = acute myocardial infarction; HF = heart failure; QALY = quality-adjusted life-year; SGLT2 = sodium-glucose cotransporter 2; SHI = statutory health insurance.

**Supplemental Table 2.** Health states and event implementation.

| **Model element** | **Implementation** | **Costs and utilities** |
| --- | --- | --- |
| Post-MI without HF | Initial health state after index myocardial infarction, without chronic HF or post-stroke morbidity | Post-MI maintenance cost and post-MI utility applied each cycle |
| Post-MI with HF | Incident chronic HF after index myocardial infarction | Post-MI maintenance cost, chronic HF maintenance cost, and chronic HF utility applied |
| Post-stroke without HF | Chronic post-stroke state without chronic HF | Post-MI maintenance cost, chronic post-stroke cost, and post-stroke utility applied |
| Post-stroke with HF | Combined chronic post-stroke and chronic HF state | Post-MI maintenance, chronic HF maintenance, chronic post-stroke cost, and combined utility applied |
| Death | Absorbing state | No further QALYs or recurring costs applied except scenario end-of-life costs |
| HF hospitalization | Transition event; may occur in chronic HF states and contributes to total HF hospitalizations | One-cycle acute event cost and utility decrement |
| Recurrent MI | Transition event; does not create a separate chronic state in the base case | One-cycle acute event cost and utility decrement |
| Acute stroke | Transition event leading to post-stroke state among survivors | Acute event cost, acute utility decrement, and chronic post-stroke costs/utilities thereafter |

HF = heart failure; MI = myocardial infarction; QALY = quality-adjusted life-year.

**Transition probability derivation**

Annual event rates and trial-period cumulative probabilities were converted to monthly transition probabilities assuming constant hazards within the relevant interval. This approach was used for short-term EMPACT-MI-derived risks and for long-term event rates where only annualized estimates were available. The conversions applied were as follows, where p is the cycle probability, r the event rate, t the cycle length in years, P the cumulative event probability over follow-up duration T, and HR and RR the hazard ratio and recurrent-event rate ratio, respectively:

*Rate-to-probability conversion:*	$p = 1 - e^{-rt}$

*Cumulative probability-to-rate conversion:*	$r = -\frac{ln(1 - P)}{T}$

*Application of a hazard ratio:*	$r_{treatment} = HR \times r_{baseline}$

*Application of a recurrent-event rate ratio:*	$r_{treatment} = RR \times r_{baseline}$

**Supplemental Table 3.** Clinical inputs and treatment-effect implementation.

| **Parameter** | **Base-case value** | **Model role** | **Distribution / scenario** | **Source** |
| --- | --- | --- | --- | --- |
| First HF hospitalization HR | 0.77 | Primary treatment effect on first HF hospitalization while receiving empagliflozin | Log-normal; 95% CI 0.60-0.98 | EMPACT-MI |
| Total HF hospitalization RR | 0.67 | Scenario treatment effect on total/recurrent HF hospitalizations | Log-normal; 95% CI 0.51-0.89 | EMPACT-MI HF analysis |
| All-cause mortality HR | 0.96 | Not applied in base case; exploratory mortality scenario | Log-normal; 95% CI 0.78-1.19 | EMPACT-MI |
| First HF hospitalization risk, placebo | 4.7% at 17.9 months | Validation target and baseline short-term risk | Beta or rate uncertainty | EMPACT-MI |
| First HF hospitalization risk, empagliflozin | 3.6% at 17.9 months | Validation check after applying treatment effect | Derived | EMPACT-MI |
| All-cause death risk, placebo | 5.5% at 17.9 months | Validation target | Beta or rate uncertainty | EMPACT-MI |
| All-cause death risk, empagliflozin | 5.2% at 17.9 months | Validation check; not used as direct base-case treatment effect | Scenario only | EMPACT-MI |
| Background mortality | Age- and sex-specific German life tables | Non-cardiovascular and background death risk | Deterministic in base case | Destatis |
| Treatment duration | 18 months | Duration of empagliflozin acquisition cost and treatment effect in current output | 18 months, 5 years, lifetime | Model assumption |

CI = confidence interval; HF = heart failure; HR = hazard ratio; RR = rate ratio.

## Utility inputs

Health-state utilities were selected from published EQ-5D-based cardiovascular, heart failure, and stroke utility literature. Utility differences between strategies arise from differences in time spent in post-MI, chronic HF, and post-stroke health states and from acute event decrements. A direct treatment-related utility gain for empagliflozin was not applied.

**Supplemental Table 4.** Utility inputs and acute disutilities.

| **Utility parameter** | **Base-case value** | **Uncertainty range used in sensitivity analysis** | **Implementation** |
| --- | --- | --- | --- |
| Post-MI without HF | 0.85 | 0.78-0.90 | Applied to post-MI without HF health state |
| Post-MI with HF | 0.76 | 0.70-0.82 | Applied to chronic HF state after MI |
| Post-stroke without HF | 0.64 | +/-20% | Applied to chronic post-stroke state |
| Post-stroke with HF | 0.57 | Scenario: multiplicative versus additive decrement | Derived using multiplicative combination of HF and post-stroke utilities |
| Acute HF hospitalization decrement | 0.194 for one cycle | +/-20% | Applied in cycles with HF hospitalization |
| Acute recurrent MI decrement | 0.130 for one cycle | +/-20% | Applied in cycles with recurrent MI |
| Acute stroke decrement | 0.290 for one cycle | +/-20% | Applied in acute stroke cycle among survivors |

Values are health-state utilities unless described as decrements. HF = heart failure; MI = myocardial infarction.

## Cost inputs

Costs were expressed in euros from the German SHI perspective. Hospital event costs were estimated using German aG-DRG relative weights and the 2026 federal base rate. Chronic costs were derived from German cardiovascular cost literature and inflated where required. Drug costs used publicly available German pharmacy prices; confidential statutory rebates were explored through price-discount scenarios.

**Supplemental Table 5.** Cost inputs used in the German SHI analysis.

| **Cost input** | **Base-case value** | **Range or scenario** | **Implementation** |
| --- | --- | --- | --- |
| Empagliflozin 10 mg once daily | €860.75/year | 25%, 50%, 75% price discount; price-threshold analysis | Applied while receiving treatment |
| HF hospitalization | €4,676/event | €1,304-€13,334; severe HF scenario €21,231 | Acute transition-event cost |
| Recurrent MI | €8,000/event | €4,700-€11,600; first-year post-MI scenario | Acute transition-event cost |
| Acute stroke | €7,572/event | €5,571-€12,001; first-year stroke scenario | Acute transition-event cost |
| Post-MI maintenance care | €3,016/year | €1,500-€4,500 | Applied in all alive states |
| Chronic HF maintenance cost | €1,157/year | €700-€2,000 | Applied in chronic HF states; excludes HF hospitalization cost to avoid double counting |
| Chronic post-stroke care | €8,424/year | +/-30% | Applied in post-stroke states |

Costs are in 2026 euros unless otherwise stated. DRG = diagnosis-related group; HF = heart failure; MI = myocardial infarction; SHI = statutory health insurance.

## Parameter uncertainty

Probabilistic sensitivity analysis used 10,000 Monte Carlo simulations. Parameter distributions were selected to respect the mathematical properties of each input. Where reported confidence intervals were unavailable, uncertainty was approximated using a +/-20% range around the base-case value.

**Supplemental Table 6.** Parameter distributions for probabilistic sensitivity analysis.

| **Parameter type** | **Distribution** | **Rationale** |
| --- | --- | --- |
| Transition probabilities | Beta | Bounded between 0 and 1 |
| Mutually exclusive transition probabilities | Dirichlet where applicable | Preserves probabilities summing to 1 |
| Utilities | Beta | Bounded between 0 and 1 |
| Costs | Gamma | Positive and right-skewed |
| Hazard ratios and rate ratios | Log-normal | Positive ratio-scale parameter |
| Discount rate | Scenario analysis | Methodological uncertainty handled outside PSA |
| Treatment duration | Scenario analysis | Structural uncertainty handled outside PSA |

PSA = probabilistic sensitivity analysis.

## Budget impact analysis assumptions

The supplementary uptake analysis was designed to specify how adoption scenarios would be operationalized from a payer-budget perspective. The eligible-population denominator used for the population-level EVPI analysis was not used to estimate a full net budget impact, because uptake, treatment duration, negotiated net prices, and indication-specific implementation remain uncertain.

**Supplemental Table 7.** Prespecified uptake scenarios for the supplementary budget impact analysis.

| **Scenario** | **Year 1** | **Year 2** | **Year 3** | **Year 4** | **Year 5** |
| --- | --- | --- | --- | --- | --- |
| Conservative | 10% | 15% | 20% | 25% | 30% |
| Moderate | 25% | 35% | 45% | 55% | 65% |
| High | 50% | 60% | 70% | 80% | 90% |
| Full adoption | 100% | 100% | 100% | 100% | 100% |

The table reports the proportion of eligible patients assumed to receive early empagliflozin in each year.

## Validation and reproducibility

**Supplemental Table 8.** Model validation and technical verification checklist.

| **Domain** | **Activity** | **Result or acceptance criterion** |
| --- | --- | --- |
| Validation | First HF hospitalization, placebo | Model 4.6% versus observed 4.7% at 17.9 months |
| Validation | First HF hospitalization, empagliflozin | Model 3.5% versus observed 3.6% at 17.9 months |
| Validation | All-cause death | Model approximately 5.6% in both arms; observed 5.5% placebo and 5.2% empagliflozin |
| Internal validity | Transition probability checks | Probabilities constrained to valid ranges; impossible transitions excluded |
| Internal validity | Cohort conservation | Alive and dead state occupancy summed to 1 in every cycle |
| Technical verification | Rate-to-probability transformations | Hand checks performed for EMPACT-MI-derived transition probabilities |
| Face validity | Clinical pathway review | State structure reviewed for clinical plausibility |
| Extreme-value testing | Zero treatment effect; zero drug cost; high event cost | Model behaviour evaluated for logical consistency |

HF = heart failure.

**Supplemental Table 9.** Planned reproducibility file structure.

| **File** | **Content** |
| --- | --- |
| parameters_clinical.csv | Clinical inputs, treatment effects, and risk parameters |
| parameters_costs.csv | Drug, event, and chronic-state cost inputs |
| parameters_utilities.csv | Health-state utilities and acute disutilities |
| model_structure.R | Health-state transition model |
| run_basecase.R | Base-case analysis |
| run_psa.R | Probabilistic sensitivity analysis |
| run_scenarios.R | Scenario and deterministic sensitivity analyses |
| run_voi.R | Expected value of perfect information analyses |
| figures_tables.R | Tables and figures for manuscript and supplement |

This reproducibility file structure should accompany the final submission package when the analytic code is archived.

# Supplemental Results

German-epidemiology base case. Background mortality was modelled with age- and sex-specific probabilities from the German period life table (Statistisches Bundesamt, Sterbetafel 2020/2022), weighted 75% male/25% female to the modelled cohort and extrapolated across the lifetime horizon by a log-linear (Gompertz) fit. A constant post-MI excess-mortality hazard was added on top of this life-table background and calibrated so that modelled first-year all-cause mortality reproduced the ~3.7% observed in contemporary, guideline-treated German post-MI survivors (OMEGA); excess mortality is therefore additive to, not a replacement for, the life-table background, precluding double counting. The baseline first heart-failure-hospitalization risk was retained from the randomized EMPACT-MI placebo arm because a targeted search of German statutory-claims and registry sources identified no rate sufficiently compatible with the EMPACT-MI-eligible (post-MI, reduced-EF or congestion) population and the HF-hospitalization endpoint; available nationwide German cohorts are unselected and substantially older, with heart failure recorded at hospitalization far more frequently than the trial's HF-hospitalization endpoint, and are reported here only as a separate real-world/high-risk scenario. The randomized treatment effect (first-HF-hospitalization HR 0.77) was unchanged. The fully trial-anchored configuration (EMPACT-MI placebo event and mortality rates) is retained as a sensitivity and validation analysis.

## Validation against EMPACT-MI

The model reproduced the observed trial-period first heart failure hospitalization risks and all-cause mortality risks from EMPACT-MI within close tolerance over the median follow-up duration of 17.9 months.

**Supplemental Table 10. Validation of modelled event risks against EMPACT-MI over 17.9 months.**

| **Outcome** | **Observed EMPACT-MI** | **Modelled** | **Absolute difference** |
| --- | --- | --- | --- |
| First HF hospitalization, placebo | 4.7% | 4.6% | -0.1 percentage points |
| First HF hospitalization, empagliflozin | 3.6% | 3.5% | -0.1 percentage points |
| All-cause death, placebo | 5.5% | 5.6% | +0.1 percentage points |
| All-cause death, empagliflozin | 5.2% | 5.6% | +0.4 percentage points |

HF = heart failure.

**Supplemental Figure 1. Model validation against EMPACT-MI.** Bars show observed EMPACT-MI event risks and modelled risks over the 17.9-month trial follow-up period. HF = heart failure.

## Cohort traces and heart failure hospitalization trajectories

Supplemental Figures 2 and 3 show modelled health-state occupancy and cumulative heart failure hospitalization trajectories over the lifetime horizon. The between-arm separation is modest, consistent with the conservative assumption of no direct mortality benefit and the relatively small absolute reduction in early heart failure hospitalization.

**Supplemental Figure 2.** Health-state cohort trace. Cohort occupancy is shown for standard care and early empagliflozin over the lifetime horizon. HF = heart failure; MI = myocardial infarction.

**Supplemental Figure 3.** Cumulative heart failure hospitalizations. The figure shows modelled cumulative heart failure hospitalizations per patient over the lifetime horizon under standard care and early empagliflozin. HF = heart failure.

## Scenario and deterministic sensitivity analyses

**Supplemental Table 11.** Scenario analyses.

| **Scenario** | **Incremental costs** | **Incremental QALYs** | **ICER** |
| --- | --- | --- | --- |
| Base case (German epidemiology) | €1,418 | 0.0315 | €45,100/QALY |
| EMPACT-MI trial-anchored (multinational placebo event and mortality rates) | €1,417 | 0.0300 | €47,200/QALY |
| Treatment duration 18 months | €1,081 | 0.0244 | €44,400/QALY |
| Treatment duration 5 years | €3,205 | 0.0653 | €49,100/QALY |
| Lifetime treatment | €7,937 | 0.1192 | €66,600/QALY |
| 25% drug price reduction | €1,016 | 0.0315 | €32,300/QALY |
| 50% drug price reduction | €613 | 0.0315 | €19,500/QALY |
| 75% drug price reduction | €211 | 0.0315 | €6,700/QALY |
| Observed mortality HR 0.96 during treatment | €1,561 | 0.0546 | €28,600/QALY |
| Low HF hospitalization cost | €1,524 | 0.0315 | €48,400/QALY |
| High HF hospitalization cost | €1,313 | 0.0315 | €41,700/QALY |
| No discounting | €1,473 | 0.0445 | €33,100/QALY |
| 5% discounting of costs and effects | €1,390 | 0.0257 | €54,100/QALY |
| No chronic HF maintenance cost increment | €1,517 | 0.0315 | €48,200/QALY |
| Larger HF utility decrement | €1,418 | 0.0400 | €35,500/QALY |

Costs are per patient. HF = heart failure; HR = hazard ratio; ICER = incremental cost-effectiveness ratio; QALY = quality-adjusted life-year.

**Supplemental Figure 4.** Scenario analyses. Bars show the ICER across prespecified scenario analyses. The solid vertical line indicates the base-case ICER; dashed and dotted lines show illustrative willingness-to-pay values. HF = heart failure; HR = hazard ratio; ICER = incremental cost-effectiveness ratio; QALY = quality-adjusted life-year.

**Supplemental Table 12.** Extended one-way deterministic sensitivity analysis.

| **Parameter** | **Low value** | **High value** | **ICER at low value** | **ICER at high value** |
| --- | --- | --- | --- | --- |
| HR first HF hospitalization | 0.60 | 0.98 | €23,175 | €586,189 |
| Empagliflozin annual cost | €430 | €1,291 | €19,493 | €70,662 |
| Utility post-MI without HF | 0.78 | 0.90 | €58,792 | €38,639 |
| Discount rate for effects | 0% | 5% | €31,891 | €55,205 |
| Utility chronic HF | 0.70 | 0.82 | €39,131 | €53,155 |
| HF hospitalization cost | €1,304 | €13,334 | €49,919 | €32,645 |
| Baseline first HF hospitalization rate | 0.027/year | 0.041/year | €53,766 | €37,518 |
| Recurrent HF hospitalization rate | 0.15/year | 0.45/year | €48,972 | €44,655 |
| Annual chronic HF maintenance cost | €700 | €2,000 | €46,312 | €42,800 |
| Annual post-MI care cost | €1,500 | €4,500 | €43,728 | €46,398 |

HF = heart failure; HR = hazard ratio; ICER = incremental cost-effectiveness ratio; MI = myocardial infarction.

## Probabilistic sensitivity analysis and value of information

**Supplemental Table 13.** Probabilistic sensitivity analysis summary.

| **Outcome** | **Mean** | **2.5th percentile** | **97.5th percentile** |
| --- | --- | --- | --- |
| Incremental costs | €1,425 | €1,197 | €1,596 |
| Incremental QALYs | 0.031 | 0.003 | 0.060 |
| Incremental life-years | 0.027 | 0.000 | 0.053 |
| HF hospitalizations avoided | 0.050 | 0.000 | 0.099 |
| Probabilistic ICER using mean costs and QALYs | €46,500/QALY | - | - |

Results are based on 10,000 Monte Carlo simulations. HF = heart failure; ICER = incremental cost-effectiveness ratio; QALY = quality-adjusted life-year.

*The probabilistic mean results shown above are consistent with the deterministic base-case estimates (main-text Table 3) and may be regarded as the base-case cost-effectiveness estimate.*

**Supplemental Table 14.** Cost-effectiveness acceptability and expected value of perfect information.

| **Willingness-to-pay value** | **Probability cost effective** | **EVPI per patient** |
| --- | --- | --- |
| €20,000/QALY | 1.8% | €3 |
| €30,000/QALY | 16.2% | €47 |
| €50,000/QALY | 55.2% | €269 |
| €80,000/QALY | 79.7% | €142 |
| €100,000/QALY | 85.8% | €111 |

Germany has no official QALY-based willingness-to-pay threshold; values are shown as illustrative analytical benchmarks. EVPI = expected value of perfect information; QALY = quality-adjusted life-year.

**Supplemental Figure 5.** Expected value of perfect information. Per-patient EVPI is shown across willingness-to-pay values from €20,000 to €100,000 per QALY gained. EVPI = expected value of perfect information; QALY = quality-adjusted life-year.

**Population-level expected value of perfect information**

To assess the decision relevance of residual uncertainty at the payer-population level, per-patient EVPI was scaled to an eligible German statutory health insurance (SHI) population over a 5-year decision horizon. The annual acute myocardial infarction (AMI) population was based on 185,400 hospitalized myocardial infarction cases in Germany. This denominator was adjusted for SHI coverage using a factor of 0.90. In the base case, 25% of AMI patients were assumed to meet EMPACT-MI-like high-risk eligibility criteria; 15% and 35% were examined as lower and upper eligibility assumptions. Future eligible cohorts were discounted at 3% annually, consistent with the base-case economic evaluation.

Population EVPI was calculated as:

Population EVPI = per-patient EVPI × annual AMI cases × SHI coverage × EMPACT-MI-like eligibility proportion × discounted 5-year population multiplier.

The discounted 5-year population multiplier was 4.717. Under the base-case eligibility assumption, the estimated annual eligible SHI population was 41,715 patients, corresponding to a discounted 5-year eligible population of 196,774 patients.

**Supplemental Table 14b.** Population-level expected value of perfect information

| **Willingness-to-pay value** | **Per-patient EVPI** | **Annual eligible SHI population** | **Discounted 5-year eligible population** | **Population EVPI** |
| --- | --- | --- | --- | --- |
| €20,000/QALY | €3 | 41,715 | 196,774 | €0.5 million |
| €30,000/QALY | €47 | 41,715 | 196,774 | €9.2 million |
| €50,000/QALY | €269 | 41,715 | 196,774 | €52.9 million |
| €80,000/QALY | €142 | 41,715 | 196,774 | €28.0 million |
| €100,000/QALY | €111 | 41,715 | 196,774 | €21.9 million |

**Supplemental Table 14c.** Sensitivity analysis for population-level EVPI at €50,000/QALY

| **EMPACT-MI-like eligibility assumption** | **Annual eligible SHI population** | **Discounted 5-year eligible population** | **Population EVPI at €50,000/QALY** |
| --- | --- | --- | --- |
| 15% of AMI patients | 25,029 | 118,064 | €31.8 million |
| 25% of AMI patients | 41,715 | 196,774 | €52.9 million |
| 35% of AMI patients | 58,401 | 275,483 | €74.1 million |

EVPI = expected value of perfect information; QALY = quality-adjusted life-year; AMI = acute myocardial infarction; SHI = statutory health insurance. Germany has no official QALY-based willingness-to-pay threshold; values are shown as illustrative analytical benchmarks. The population-level EVPI analysis should be interpreted as an exploratory scaling exercise because the future eligible population for a hypothetical post-MI heart failure prevention indication remains uncertain.

**Exploratory European cross-country cost-effectiveness**

As an exploratory extension beyond the German base case, the EMPACT-MI clinical effect was applied across the EU-27 using country-specific empagliflozin list prices, country-specific heart failure hospitalization costs, and gross domestic product (GDP)-based willingness-to-pay thresholds (1× and 3× GDP per capita). The incremental QALY gain (0.0300, trial-anchored EMPACT-MI configuration) was held constant; only prices and heart failure hospitalization costs were varied by country. Value was strongly price-driven: the ICER ranged from approximately €17,600/QALY (France) to €46,800/QALY (Germany, reproducing the base case), and early empagliflozin was cost-effective at 1× GDP per capita in higher-price/higher-income countries only.

**Supplemental Table 14d.** Exploratory cost-effectiveness of early empagliflozin across EU-27 countries at national list prices and GDP-based willingness-to-pay thresholds.

| **Country** | **Annual empagliflozin cost (€)** | **Incremental cost (€)** | **ICER (€/QALY)** | **WTP 1×GDP (€)** | **Probability CE at 1×GDP** | **Probability CE at 3×GDP** |
| --- | --- | --- | --- | --- | --- | --- |
| France | €445 | €524 | €17,584 | €41,340 | 90% | 100% |
| Sweden | €502 | €555 | €18,619 | €50,490 | 93% | 100% |
| Netherlands | €508 | €572 | €19,207 | €58,740 | 95% | 100% |
| Slovenia | €412 | €598 | €20,057 | €30,200 | 72% | 99% |
| Lithuania | €392 | €619 | €20,771 | €25,880 | 61% | 97% |
| Estonia | €417 | €656 | €21,997 | €28,080 | 62% | 98% |
| Luxembourg | €606 | €691 | €23,201 | €122,970 | 99% | 100% |
| Poland | €442 | €751 | €25,188 | €19,980 | 28% | 90% |
| Ireland | €626 | €767 | €25,749 | €99,080 | 98% | 100% |
| Austria | €625 | €797 | €26,742 | €52,330 | 84% | 99% |
| Romania | €461 | €806 | €27,036 | €16,870 | 14% | 82% |
| Belgium | €624 | €820 | €27,501 | €51,090 | 82% | 99% |
| Hungary | €492 | €844 | €28,333 | €20,540 | 22% | 88% |
| Slovakia | €507 | €845 | €28,352 | €22,640 | 28% | 90% |
| Portugal | €553 | €870 | €29,204 | €25,560 | 35% | 93% |
| Spain | €600 | €938 | €31,466 | €30,980 | 43% | 95% |
| Bulgaria | €539 | €941 | €31,575 | €14,660 | 4% | 68% |
| Finland | €674 | €949 | €31,852 | €48,950 | 73% | 99% |
| Greece | €568 | €952 | €31,943 | €21,300 | 18% | 85% |
| Cyprus | €623 | €994 | €33,350 | €33,870 | 46% | 95% |
| Latvia | €607 | €1,033 | €34,650 | €21,030 | 13% | 81% |
| Czechia | €634 | €1,035 | €34,733 | €29,330 | 33% | 92% |
| Denmark | €773 | €1,035 | €34,739 | €62,910 | 81% | 99% |
| Croatia | €707 | €1,239 | €41,584 | €20,530 | 5% | 71% |
| Italy | €773 | €1,246 | €41,812 | €36,330 | 34% | 92% |
| **Germany** | **€957** | **€1,395** | **€46,812** | **€50,660** | **50%** | **96%** |

Exploratory analysis reusing an EU-27 scaffold (country-specific heart failure hospitalization costs and 1×/3× GDP-per-capita willingness-to-pay values). The clinical effect (0.0300 QALYs, trial-anchored EMPACT-MI configuration) was held constant from EMPACT-MI; only country-specific empagliflozin list prices (public national list/pharmacy price per 30-tablet pack, annualized) and heart failure hospitalization costs were varied. Confidential negotiated net prices may differ from list prices. GDP-based thresholds are illustrative analytical benchmarks, not official willingness-to-pay thresholds. Malta was omitted because no public price could be located. CE = cost-effective; GDP = gross domestic product; ICER = incremental cost-effectiveness ratio; P(CE) = probability cost-effective; QALY = quality-adjusted life-year; WTP = willingness-to-pay.

**Supplemental Table 15.** Trial and economic evidence used for model anchoring and contextualisation.

| **Evidence source** | **Population / comparison** | **Key results relevant to this model** | **Use in current economic analysis** |
| --- | --- | --- | --- |
| EMPACT-MI primary trial (Butler et al.) | 6522 patients hospitalized for acute MI and at increased risk of HF; empagliflozin 10 mg versus placebo | Primary composite of first HHF or all-cause death: HR 0.90 (95% CI 0.76-1.06). First HHF: HR 0.77 (95% CI 0.60-0.98). All-cause death: HR 0.96 (95% CI 0.78-1.19). | Primary source for base-case treatment effect on first HHF; no direct mortality benefit applied in base case. |
| EMPACT-MI HF outcomes analysis (Hernandez et al.) | Prespecified HF outcomes analysis within EMPACT-MI | First HHF 118 versus 153; total HHF 148 versus 207; total HHF RR 0.67 (95% CI 0.51-0.89). | Source for recurrent/total HHF scenario analyses and validation of HF event reduction. |
| EMPACT-MI T2D/CKD subgroup analysis (Fioretti et al.) | Patients after acute MI with and without type 2 diabetes and/or chronic kidney disease | Total HHF RR 0.75 (95% CI 0.48-1.18) without T2D/CKD and RR 0.65 (95% CI 0.45-0.94) with T2D/CKD; P interaction = 0.61. | Supports assumption of broadly consistent HF-event effect across clinically relevant subgroups; not used to modify base-case treatment effect. |
| DAPA-MI primary trial (James et al.) | 4017 patients after acute MI without prior diabetes or chronic HF; dapagliflozin 10 mg versus placebo | Win ratio for hierarchical composite 1.34 (95% CI 1.20-1.50); cardiovascular death/HHF HR 0.95 (95% CI 0.64-1.40). | Contextual evidence for post-MI SGLT2 inhibition; not used as a base-case empagliflozin treatment-effect input. |
| DAPA-MI LVEF analysis (Erlinge et al.) | DAPA-MI patients stratified by baseline LVEF <50% versus >=50% | Primary hierarchical composite favoured dapagliflozin in both LVEF strata; no significant interaction and no effect on cardiovascular death/HHF. | Supports external contextualisation and class-effect discussion; not used for base-case transition probabilities. |
| Economic model in HFpEF (Zheng et al.) | Markov cost-effectiveness model of empagliflozin in HFpEF | Empagliflozin cost-effectiveness was highly sensitive to drug price, quality-of-life benefit, and mortality assumptions. | Informed selection of price, utility, and mortality scenario analyses. |

AMI = acute myocardial infarction; CI = confidence interval; CKD = chronic kidney disease; HF = heart failure; HFpEF = heart failure with preserved ejection fraction; HHF = hospitalization for heart failure; HR = hazard ratio; LVEF = left ventricular ejection fraction; MI = myocardial infarction; RR = rate ratio; SGLT2 = sodium-glucose cotransporter 2; T2D = type 2 diabetes.

**Supplemental Table 16.** Detailed base-case economic result tie-out.

| **Outcome** | **Standard care** | **Early empagliflozin** | **Increment** | **Interpretation** |
| --- | --- | --- | --- | --- |
| Discounted costs, € | 47,520 | 48,939 | 1,418 | Incremental costs driven primarily by empagliflozin acquisition. |
| Discounted QALYs | 8.321 | 8.352 | 0.0315 | Quality-adjusted survival gain driven by delayed HF progression and fewer HF hospitalizations. |
| Discounted life-years | 10.193 | 10.221 | 0.028 | No direct mortality effect was applied; life-year gain is therefore modest. |
| ICER, €/QALY | - | - | 45,100 | Rounded to nearest €100 in the main manuscript. |
| INMB at €30,000/QALY, € | - | - | −474 | Negative net benefit at illustrative €30,000/QALY. |
| INMB at €50,000/QALY, € | - | - | 155 | Approximately neutral at illustrative €50,000/QALY. |
| INMB at €80,000/QALY, € | - | - | 1,099 | Positive net benefit at illustrative €80,000/QALY. |
| INMB at €100,000/QALY, € | - | - | 1,728 | Positive net benefit at illustrative €100,000/QALY. |

ICER = incremental cost-effectiveness ratio; INMB = incremental net monetary benefit; QALY = quality-adjusted life-year. Values may not sum exactly because calculations used unrounded model outputs.

**Supplemental Table 17.** Modelled lifetime clinical outcomes.

| **Clinical outcome** | **Standard care per 1000 patients** | **Early empagliflozin per 1000 patients** | **Difference per 1000 patients** | **Model interpretation** |
| --- | --- | --- | --- | --- |
| First HF hospitalization / incident HF | 342.4 | 333.0 | −9.5 | Fewer transitions from post-MI without HF to chronic HF. |
| Recurrent HF hospitalizations | 1140.0 | 1097.5 | −42.5 | Fewer recurrent HF events in patients with chronic HF. |
| Total HF hospitalizations | 1482.4 | 1430.5 | −52.0 | Primary modelled clinical benefit. |
| Recurrent MI events | 394.1 | 395.3 | +1.2 | No direct treatment effect on recurrent MI applied. |
| Stroke events | 181.5 | 182.1 | +0.6 | No direct treatment effect on stroke applied; small difference reflects survival/time-at-risk. |
| Deaths | 999.9 | 999.9 | 0.0 | No direct mortality benefit applied; all patients eventually die over lifetime horizon. |

HF = heart failure; MI = myocardial infarction. The table reports lifetime cumulative events per 1000 patients entering the model.

**Supplemental Table 18.** Discounted life-years and QALYs by health-state component.

| **Outcome component** | **Standard care LYs** | **Empagliflozin LYs** | **Delta LYs** | **Standard care QALYs** | **Empagliflozin QALYs** | **Delta QALYs** |
| --- | --- | --- | --- | --- | --- | --- |
| Post-MI without HF | 7.756 | 7.861 | 0.105 | 6.593 | 6.682 | 0.089 |
| Post-MI with HF | 1.708 | 1.628 | −0.080 | 1.298 | 1.237 | −0.061 |
| Post-stroke without HF | 0.544 | 0.552 | 0.008 | 0.348 | 0.353 | 0.005 |
| Post-stroke with HF | 0.185 | 0.179 | −0.005 | 0.105 | 0.102 | −0.003 |
| Acute event disutilities | — | — | — | −0.024 | −0.023 | 0.001 |
| Total | 10.193 | 10.221 | 0.028 | 8.321 | 8.352 | 0.0315 |

HF = heart failure; LY = life-year; MI = myocardial infarction; QALY = quality-adjusted life-year. Acute event disutility reconciles state-based QALYs with total QALYs and reflects temporary decrements for acute events.

**Supplemental Table 19.** Detailed discounted lifetime cost components.

| **Cost component** | **Standard care, €** | **Early empagliflozin, €** | **Increment, €** | **Comment** |
| --- | --- | --- | --- | --- |
| Empagliflozin acquisition | 0 | 1,610 | 1,610 | Applied during treatment duration. |
| Routine post-MI care | 30,741 | 30,827 | 86 | Small increase due to additional survival/time at risk. |
| Chronic HF maintenance | 2,190 | 2,091 | −98 | Lower time spent with chronic HF. |
| HF hospitalizations | 4,932 | 4,720 | −211 | Event cost offset from fewer HF hospitalizations. |
| Recurrent MI events | 2,443 | 2,450 | 3 | No direct treatment effect applied. |
| Stroke events | 1,074 | 1,077 | 2 | No direct treatment effect applied. |
| Chronic post-stroke care | 6,140 | 6,164 | 24 | Slightly higher due to increased survival/time at risk. |
| Total costs | 47,520 | 48,939 | 1,418 | Direct medical costs from German SHI perspective. |

€ = euros; HF = heart failure; MI = myocardial infarction; SHI = statutory health insurance.

**Supplemental Table 20.** Drug price and price-threshold analyses.

| **Price scenario** | **Annual empagliflozin cost, €** | **Incremental costs, €** | **Incremental QALYs** | **ICER or threshold result** |
| --- | --- | --- | --- | --- |
| Current German public pharmacy price | 860.75 | 1,418 | 0.0315 | €45,100/QALY |
| 25% price reduction | 645.56 | 1,016 | 0.0315 | €32,300/QALY |
| 50% price reduction | 430.38 | 613 | 0.0315 | €19,500/QALY |
| 75% price reduction | 215.19 | 211 | 0.0315 | €6,700/QALY |
| Maximum annual price at €30,000/QALY | 606.94 | - | - | Approx. 29% reduction versus current price |
| Maximum annual price at €50,000/QALY | 944.02 | - | - | Slightly above current modelled price |
| Maximum annual price at €80,000/QALY | 1,448.00 | - | - | Threshold-derived upper price |
| Maximum annual price at €100,000/QALY | 1,785.00 | - | - | Threshold-derived upper price |

ICER = incremental cost-effectiveness ratio; QALY = quality-adjusted life-year. Germany has no official QALY-based willingness-to-pay threshold; threshold values are illustrative analytical benchmarks.

## Budget impact uptake assumptions

The uptake scenarios below support future budget impact analyses. A full net monetary budget impact was not reported because uptake, treatment duration, and negotiated net prices remain uncertain; the eligible-population denominator estimated for the population-level value-of-information analysis (Supplemental Tables 14b and 14c) could, however, inform such analyses.

**Supplemental Figure 6.** Budget impact uptake scenarios. The figure shows the proportion of eligible patients assumed to receive early empagliflozin in each budget impact year under conservative, moderate, high, and full-adoption scenarios.

**Supplemental Table 21.** CHEERS 2022 reporting checklist.

| **Item** | **CHEERS 2022 item** | **Location in manuscript / supplement** | **Reported information** |
| --- | --- | --- | --- |
| 1 | Title | Title page; Abstract | Title identifies a cost-effectiveness/cost-utility evaluation of early empagliflozin after acute myocardial infarction from the German statutory health insurance perspective. |
| 2 | Abstract | Abstract | Structured summary reports objective, model, perspective, QALYs, costs, ICER, uncertainty, price thresholds, and conclusion. |
| 3 | Background and objectives | Introduction | Clinical and economic burden of post-MI heart failure, EMPACT-MI evidence, German reimbursement context, and study objective are described. |
| 4 | Health economic analysis plan | Methods - Study Design and Reporting | No separate health economic analysis plan was prospectively registered; model structure, inputs, assumptions, scenarios, and validation are reported in the manuscript and supplement. |
| 5 | Study population | Methods - Population, Intervention, and Comparator; Table 1 | Adults hospitalized for acute myocardial infarction and at increased risk of heart failure, reflecting EMPACT-MI eligibility; prespecified high-risk subgroups are listed. |
| 6 | Setting and location | Methods - Study Design and Reporting; Costs | Germany; German statutory health insurance setting; direct medical costs included. |
| 7 | Comparators | Methods - Population, Intervention, and Comparator; Table 1 | Empagliflozin 10 mg once daily plus contemporary standard care versus standard post-MI care without systematic early sodium-glucose cotransporter 2 inhibitor initiation; delayed sodium-glucose cotransporter 2 inhibitor use after heart failure onset allowed. |
| 8 | Perspective | Methods - Study Design and Reporting; Table 1 | German statutory health insurance perspective with direct medical costs only. |
| 9 | Time horizon | Methods - Model Structure; Table 1 | Lifetime horizon until death or age 100 years, selected to capture downstream heart failure and cardiovascular consequences beyond trial follow-up. |
| 10 | Discount rate | Methods - Discounting, Outcomes, and Cost-Effectiveness Analysis; Table 1 | Costs and health outcomes discounted at 3% annually in the base case; 0%, 5%, and differential discounting explored. |
| 11 | Selection of outcomes | Methods - Discounting, Outcomes, and Cost-Effectiveness Analysis; Table 1 | Outcomes include QALYs, life-years, costs, ICERs, incremental net monetary benefit, heart failure hospitalizations, recurrent MI, stroke, deaths, CEACs, and value of information. |
| 12 | Measurement of outcomes | Methods - Clinical Effectiveness and Transition Probabilities; Supplemental Methods | Outcomes generated by the cohort state-transition model using EMPACT-MI event risks/effects, German life tables, and published cardiovascular evidence. |
| 13 | Valuation of outcomes | Methods - Health Outcomes and Utilities; Supplemental Table 4 | EQ-5D-based utilities assigned to post-MI, chronic heart failure, post-stroke, and combined states; acute disutilities applied for heart failure hospitalization, recurrent MI, and stroke. |
| 14 | Measurement and valuation of resources and costs | Methods - Costs; Supplemental Table 5 | Drug acquisition, routine post-MI care, chronic heart failure care, heart failure hospitalization, recurrent MI, stroke, and post-stroke costs valued using German sources. |
| 15 | Currency, price date, and conversion | Methods - Costs; Tables 1-2; Supplemental Table 5 | Costs reported in 2026 euros; German public pharmacy prices, 2026 aG-DRG tariffs, and 2026 federal base rate used; older costs inflated where required. |
| 16 | Rationale and description of model | Methods - Model Structure; Figure 1; Supplemental Tables 1-2 | Cohort state-transition Markov model with monthly cycles, chronic health states, acute transition events, half-cycle correction, and simplified structure justified for transparent lifetime extrapolation. |
| 17 | Analytics and assumptions | Methods; Supplemental Methods; Supplemental Tables 3, 6, and 8 | Rate-to-probability conversions, treatment-effect implementation, no direct mortality benefit in the base case, distributions for PSA, scenario analyses, and validation checks are reported. |
| 18 | Characterizing heterogeneity | Methods - Subgroup and Heterogeneity Analyses | Prespecified subgroup analyses evaluate age >=65 years, type 2 diabetes or chronic kidney disease, no type 2 diabetes or chronic kidney disease, LVEF <35%, and congestion during index hospitalization. |
| 19 | Characterizing distributional effects | Methods - Subgroup and Heterogeneity Analyses; Discussion | Formal distributional cost-effectiveness analysis, equity weighting, and priority-population adjustment were not undertaken; heterogeneity was explored through clinical subgroup and scenario analyses. |
| 20 | Characterizing uncertainty | Methods - Uncertainty, Scenario, and Threshold Analyses; Results; Supplemental Tables 11-14 | Deterministic sensitivity analysis, PSA, structural scenarios, price-threshold analyses, CEACs, EVPI, and EVPPI are described and reported. |
| 21 | Engagement with patients and others affected by the study | Methods - Study Design and Reporting; Declarations | No formal patient, public, or stakeholder engagement process was conducted; the clinical and payer-relevant framing was informed by multidisciplinary clinical and health-economic expertise. |
| 22 | Study parameters | Table 2; Supplemental Tables 3-6 | Clinical effects, baseline risks, costs, utilities, mortality inputs, uncertainty ranges, distributions, and sources are tabulated. |
| 23 | Summary of main results | Results; Tables 3-5; Supplemental Tables 16-20 | Total and incremental costs, QALYs, life-years, ICER, incremental net monetary benefit, heart failure hospitalizations avoided, cost components, and price thresholds are reported. |
| 24 | Effect of uncertainty | Results; Figures 2-3; Supplemental Tables 11-14 | Results of one-way sensitivity analysis, PSA, scenario analyses, cost-effectiveness acceptability, value-of-information analysis, and threshold analyses are reported. |
| 25 | Effect of engagement with patients and others affected by the study | Methods - Study Design and Reporting; Declarations | Not applicable; because no formal engagement process was undertaken, no engagement-driven changes to model structure, inputs, assumptions, or interpretation were recorded. |
| 26 | Study findings, limitations, generalizability, and current knowledge | Discussion | Findings are interpreted in relation to EMPACT-MI, DAPA-MI, broader sodium-glucose cotransporter 2 inhibitor evidence, German reimbursement and pricing, patient selection, uncertainty, strengths, and limitations. |
| 27 | Source of funding | Declarations - Funding; Acknowledgements | No specific funding was received for this study. The ESC Academy educational scholarship for PB's participation in the Executive MSc programme at the London School of Economics and Political Science should be disclosed; the sponsor had no role in the study. |
| 28 | Conflicts of interest | Declarations - Competing interests | Authors should declare no relevant financial or non-financial competing interests, if confirmed before submission. |

AMI = acute myocardial infarction; CEAC = cost-effectiveness acceptability curve; CHEERS = Consolidated Health Economic Evaluation Reporting Standards; DSA = deterministic sensitivity analysis; EVPI/EVPPI = expected value of perfect/partial perfect information; HF = heart failure; ICER = incremental cost-effectiveness ratio; LVEF = left ventricular ejection fraction; MI = myocardial infarction; PSA = probabilistic sensitivity analysis; QALY = quality-adjusted life-year; SHI = statutory health insurance.

# Supplemental analysis: European cross-country price scenario

Because empagliflozin acquisition cost was a major driver of the ICER, we performed an exploratory European cross-country price scenario. For each EU member state with a verifiable public retail price, the published public pharmacy price per 30 tablets was substituted into the drug-cost input of the German base-case model, while the EMPACT-MI-derived incremental QALY gain was held constant at 0.0300 (trial-anchored configuration). Country-specific heart failure hospitalization costs and GDP-based reference values were taken from the DIGIT-HF EU27 scaffold; Germany reproduced the base-case ICER (€46,812/QALY). Malta was excluded because no verifiable public retail price was available.

This cross-country scenario is an exploratory price and net-price-threshold illustration, not a country-specific cost-effectiveness analysis. It varied country-specific drug prices, heart failure hospitalization costs and willingness-to-pay, but held the clinical event rates and mortality constant at the EMPACT-MI-derived values; a fully country-adapted analysis would additionally require national epidemiological data, unit costs and jurisdiction-specific methodological choices, and could yield different conclusions. The 1x and 3x GDP-per-capita reference values are shown for orientation only; most European jurisdictions do not anchor cost-effectiveness thresholds to GDP, and the former WHO GDP-multiple recommendation is no longer considered good practice. The scenario should therefore be interpreted only as an indication of how strongly value depends on net price across settings.

**Supplemental Figure 9.** European cross-country cost-effectiveness scenario for early empagliflozin after acute myocardial infarction. Stylised EU tile map showing country-level ICERs when national empagliflozin prices and heart failure hospitalization costs are applied while holding the EMPACT-MI clinical effect constant. Border colour indicates position relative to a 1x GDP-per-capita benchmark (illustrative only). Malta was not assessed (no verifiable public price). This exploratory price/threshold illustration is not a country-specific cost-effectiveness analysis.

Across the 26 priced member states, the price per 30 tablets ranged from €32.22 in Lithuania to €78.62 in Germany, and the corresponding ICER ranged from €17,584 to €46,812 per QALY gained. ICERs were below the illustrative 1× GDP-per-capita benchmark in 14 of 26 countries and below the 3× GDP-per-capita benchmark in all 26 countries. Lower-price markets such as France, Lithuania, and Estonia were below the 1× GDP benchmark, whereas several countries exceeded this benchmark despite lower prices than Germany. These findings indicate that cross-country conclusions were shaped not only by acquisition price but also by avoidable heart failure hospitalization costs and GDP-based affordability benchmarks.

**Supplemental Table 22.** European cross-country empagliflozin price scenario. For each member state the public pharmacy price per 30 tablets is substituted into the drug-cost input; incremental QALY gain is held constant. ICER = incremental cost-effectiveness ratio; WTP = willingness-to-pay; GDP = gross domestic product.

| **Country** | **Price per 30 tab (€)** | **Incremental cost (€)** | **ICER (€/QALY)** | **1× / 3× GDP benchmark (€/QALY)** | **ICER below 3× GDP benchmark** | **ICER below 3× GDP benchmark** |
| --- | --- | --- | --- | --- | --- | --- |
| France | 36.59 | 524 | 17,584 | 41,340 / 124,020 | Yes | Yes |
| Sweden | 41.21 | 555 | 18,619 | 50,490 / 151,470 | Yes | Yes |
| Netherlands | 41.74 | 572 | 19,207 | 58,740 / 176,220 | Yes | Yes |
| Slovenia | 33.86 | 598 | 20,057 | 30,200 / 90,600 | Yes | Yes |
| Lithuania | 32.22 | 619 | 20,771 | 25,880 / 77,640 | Yes | Yes |
| Estonia | 34.26 | 656 | 21,997 | 28,080 / 84,240 | Yes | Yes |
| Luxembourg | 49.79 | 691 | 23,201 | 122,970 / 368,910 | Yes | Yes |
| Poland | 36.32 | 751 | 25,188 | 19,980 / 59,940 | No | Yes |
| Ireland | 51.42 | 767 | 25,749 | 99,080 / 297,240 | Yes | Yes |
| Austria | 51.37 | 797 | 26,742 | 52,330 / 156,990 | Yes | Yes |
| Romania | 37.83 | 806 | 27,036 | 16,870 / 50,610 | No | Yes |
| Belgium | 51.24 | 820 | 27,501 | 51,090 / 153,270 | Yes | Yes |
| Hungary | 40.45 | 844 | 28,333 | 20,540 / 61,620 | No | Yes |
| Slovakia | 41.63 | 845 | 28,352 | 22,640 / 67,920 | No | Yes |
| Portugal | 45.40 | 870 | 29,204 | 25,560 / 76,680 | No | Yes |
| Spain | 49.31 | 938 | 31,466 | 30,980 / 92,940 | No | Yes |
| Bulgaria | 44.23 | 941 | 31,575 | 14,660 / 43,980 | No | Yes |
| Finland | 55.39 | 949 | 31,852 | 48,950 / 146,850 | Yes | Yes |
| Greece | 46.68 | 952 | 31,943 | 21,300 / 63,900 | No | Yes |
| Cyprus | 51.19 | 994 | 33,350 | 33,870 / 101,610 | Yes | Yes |
| Latvia | 49.84 | 1,033 | 34,650 | 21,030 / 63,090 | No | Yes |
| Czechia | 52.08 | 1,035 | 34,733 | 29,330 / 87,990 | No | Yes |
| Denmark | 63.51 | 1,035 | 34,739 | 62,910 / 188,730 | Yes | Yes |
| Croatia | 58.07 | 1,239 | 41,584 | 20,530 / 61,590 | No | Yes |
| Italy | 63.45 | 1,246 | 41,812 | 36,330 / 108,990 | No | Yes |
| Germany | 78.62 | 1,395 | 46,812 | 50,660 / 151,980 | Yes | Yes |

**Supplemental Figure 7.** European cross-country price scenario: actual empagliflozin price per 30 tablets versus the maximum cost-effective price at 1× GDP per capita per QALY. Countries are ordered by headroom; blue denotes cost-effective and red not cost-effective at 1× GDP. GDP = gross domestic product.

**Supplemental Figure 8.** Country-level incremental cost-effectiveness ratio (ICER) versus willingness-to-pay at 1× GDP per capita per QALY. Filled circles show the ICER and diamonds the national willingness-to-pay threshold; blue denotes cost-effective and red not cost-effective. GDP = gross domestic product.

# Supplemental References

1. Anker SD, Butler J, Filippatos G, et al. Empagliflozin in heart failure with a preserved ejection fraction. New England Journal of Medicine. 2021;385:1451-1461.

2. Betts KA, Song J, Wade RL, et al. Utility values and disutilities associated with cardiovascular events and treatment-related adverse events: a systematic literature review. Health and Quality of Life Outcomes. 2020;18:1-16.

3. Briggs AH, Sculpher MJ, Claxton K. Decision Modelling for Health Economic Evaluation. Oxford: Oxford University Press; 2006.

4. Butler J, Jones WS, Udell JA, et al. Empagliflozin after acute myocardial infarction. New England Journal of Medicine. 2024;390:1455-1466.

5. Destatis. Life tables for Germany. Wiesbaden: Federal Statistical Office of Germany.

6. Di Tanna GL, Bychenkova A, ONeill F, et al. Evaluating cost-effectiveness models for pharmacologic interventions in adults with heart failure: a systematic literature review. Pharmacoeconomics. 2021;39:1135-1156.

7. Dyer MT, Goldsmith KA, Sharples LS, Buxton MJ. A review of health utilities using the EQ-5D in studies of cardiovascular disease. Health and Quality of Life Outcomes. 2010;8:13.

8. GKV-Spitzenverband. Vereinbarung zum Bundesbasisfallwert 2026. Berlin: GKV-Spitzenverband; 2026.

9. Hernandez AF, Udell JA, Jones WS, et al. Effect of empagliflozin on heart failure outcomes after acute myocardial infarction: insights from the EMPACT-MI trial. Circulation. 2024;149:1627-1638.

10. InEK. DRG-Fallpauschalen-Katalog 2026 und Pflegeerlöskatalog 2026. Siegburg: Institut für das Entgeltsystem im Krankenhaus; 2025.

11. Joundi RA, Adekanye J, Leung AA, et al. Health state utility values in people with stroke: a systematic review and meta-analysis. Journal of the American Heart Association. 2022;11:e024296.

12. Peters-Klimm F, Halmer A, Flessa S, Szecsenyi J, Ose D. What drives the costs of heart failure care in Germany? Journal of Public Health. 2012;20:653-660.

13. Schmid T. Costs of treating cardiovascular events in Germany: a systematic literature review. Health Economics Review. 2015;5:27.

14. Zheng J, Parizo JT, Spertus JA, Heidenreich PA, Sandhu AT. Cost-effectiveness of empagliflozin in patients with heart failure with preserved ejection fraction. JAMA Internal Medicine. 2022;182:1278-1288.

15. James S, Erlinge D, Storey RF, et al. Dapagliflozin in myocardial infarction without diabetes or heart failure. NEJM Evidence. 2024;3:EVIDoa2300286.

16. Fioretti F, Butler J, Udell JA, et al. Empagliflozin after myocardial infarction with or without diabetes and chronic kidney disease: insights from EMPACT-MI. ESC Heart Failure. 2025;12:3940-3952.

17. Erlinge D, James S, Deanfield J, et al. Cardiometabolic outcomes with dapagliflozin after myocardial infarction by baseline ejection fraction: DAPA-MI. ESC Heart Failure. 2025;12:4184-4193.

18. Wissenschaftliches Institut der AOK (WIdO). Gesundheitsatlas Deutschland: Herzinfarkt – Häufigkeit stationär behandelter Fälle. Berlin: WIdO; 2024.

19. Bundesministerium für Gesundheit (BMG). Mitglieder und Versicherte der gesetzlichen Krankenversicherung (KM6-Statistik: Versicherte zum 1. Juli 2024; rund 74,5 Millionen Versicherte, etwa 89 % der Bevölkerung). Berlin: BMG; 2024.
